# Using landscape genomics to assess local adaptation and genomic vulnerability of a perennial herb *Tetrastigma hemsleyanum* (Vitaceae) in subtropical China

**DOI:** 10.3389/fgene.2023.1150704

**Published:** 2023-04-18

**Authors:** Yihan Wang, Lin Zhang, Yuchao Zhou, Wenxin Ma, Manyu Li, Peng Guo, Li Feng, Chengxin Fu

**Affiliations:** ^1^ College of Life Sciences, Henan Agricultural University, Zhengzhou, China; ^2^ Henan Engineering Research Center for Osmanthus Germplasm Innovation and Resource Utilization, Henan Agricultural University, Zhengzhou, China; ^3^ College of Landscape Architecture and Art, Henan Agricultural University, Zhengzhou, China; ^4^ School of Pharmacy, Xi’an Jiaotong University, Xi’an, China; ^5^ Key Laboratory of Conservation Biology for Endangered Wildlife of the Ministry of Education, College of Life Sciences, Zhejiang University, Hangzhou, China

**Keywords:** candidate genes, climate change, genomic variation, genomic vulnerability, local adaptation, *T. hemsleyanum*

## Abstract

Understanding adaptive genetic variation of plant populations and their vulnerabilities to climate change are critical to preserve biodiversity and subsequent management interventions. To this end, landscape genomics may represent a cost-efficient approach for investigating molecular signatures underlying local adaptation. *Tetrastigma hemsleyanum* is, in its native habitat, a widespread perennial herb of warm-temperate evergreen forest in subtropical China. Its ecological and medicinal values constitute a significant revenue for local human populations and ecosystem. Using 30,252 single nucleotide polymorphisms (SNPs) derived from reduced-representation genome sequencing in 156 samples from 24 sites, we conducted a landscape genomics study of the *T. hemsleyanum* to elucidate its genomic variation across multiple climate gradients and genomic vulnerability to future climate change. Multivariate methods identified that climatic variation explained more genomic variation than that of geographical distance, which implied that local adaptation to heterogeneous environment might represent an important source of genomic variation. Among these climate variables, winter precipitation was the strongest predictor of the contemporary genetic structure. *F*_ST_ outlier tests and environment association analysis totally identified 275 candidate adaptive SNPs along the genetic and environmental gradients. SNP annotations of these putatively adaptive loci uncovered gene functions associated with modulating flowering time and regulating plant response to abiotic stresses, which have implications for breeding and other special agricultural aims on the basis of these selection signatures. Critically, modelling revealed that the high genomic vulnerability of our focal species via a mismatch between current and future genotype-environment relationships located in central-northern region of the *T. hemsleyanum*’s range, where populations require proactive management efforts such as assistant adaptation to cope with ongoing climate change. Taken together, our results provide robust evidence of local climate adaption for *T. hemsleyanum* and further deepen our understanding of adaptation basis of herbs in subtropical China.

## 1 Introduction

Local adaptation is ubiquitous in plants, which can result in the genetic divergence of populations cross the landscape (Sork et al., 2016; Cao et al., 2020; Shen et al., 2022). Climate is a major driver of such variation (Rehfeldt et al., 2014; Jia et al., 2020; Capblancq et al., 2022), whereas the primary climatic agents of selection and targets remain unknown for many species. The widely distributed species can span multiple climatic and topographic gradients whereby both adaptive and neutral processes can both affect their genome-wide variations (Savolainen et al., 2013; Wang et al., 2015; Nadeau et al., 2016). Disentangling the relative effects of natural selection and spatial isolation on genomic variation is important to quantify the contribution of adaptation in shaping the diversification and to unravel the specific climate selective agents underpinning its appearance (Gibson and Moyle, 2020). This, however, remains technically challenging mainly because selective climatic gradients and spatial variables are often confounding in natural populations (Wiens, 1989; Kissoudis et al., 2016; Nadeau et al., 2016). Advances in evolutionary and landscape genomics approaches and the increasing accessibility of large genomic and climate datasets enabled the characterization of the independent contributions of climate and space to explaining patterns of genetic variation (Feng and Du, 2022). These strategies serve as a complement to traditional approaches uncovering evidence for local adaptation (i.e., common garden experiments and/or reciprocal transplant), and aid efforts to identify agents of selection acting in natural populations and their possible genetic targets (Lasky et al., 2012; Lu et al., 2019; Capblancq et al., 2022).

Global climate change is a significant threat to biodiversity, which is impacting biosphere and altering ecosystem functions (Capblancq et al., 2020; Malhi et al., 2020). Numerous empirical studies have demonstrated that many plant species are already affected by rapid climate change and, as a result, displaying various responses, including shifting their ranges, loss of genetic diversity and fitness in nature populations as well as changes in population genetic composition (Exposito-Alonso et al., 2019; Exposito-Alonso et al., 2022; Sang et al., 2022). To date, predicting impacts of climate change on plant species traditionally depends on species distribution modelling (SDM) (Peterson et al., 2012; Dyderski et al., 2018; Thuiller et al., 2019). Although these methods can identify species vulnerable regions to future climate changes and forecast global patterns of extinction risk via integration of species occurrence records and fine-scale climate data, the SDM assumes that all individuals within a species have similar climate stress and ignores ecotypes and local adaptation (Smith et al., 2019; Capblancq et al., 2020; Aguirre-Liguori et al., 2021). Hence, it has been criticized for oversimplification and gradually replaced by methodologies that integrated local adaptation into projecting species responses to climate change (Fitzpatrick and Keller, 2015; Exposito-Alonso et al., 2018; Exposito-Alonso et al., 2019). In addition, the predominant methods utilized for detecting putatively adaptive signatures in natural systems are *F*_ST_ outlier analyses (OA) and environmental association analysis (EAA). OA studies screen SNPs with higher genetic divergence than expected among populations under a neutral model (Hohenlohe et al., 2010; Lotterhos and Whitlock, 2015), while EAA integrates genetic variation or allele frequencies and environmental variables and then detects adaptive signatures through identifying associations between them (Frichot and François, 2015; Rellstab et al., 2015). However, to identify loci putatively under selection is but one part of the question, additional efforts are needed to unravel how the selection stress acts on associated loci along the climatic gradients, and to understand functional significance of these loci. Keeping this in mind, we utilized integrative methods, including three OAs and one EAA, followed by SNP-specific generalized dissimilarity modelling (GDM) to determine changes of adaptive allelic frequencies throughout the species range (hereafter “allelic turnover”; Fitzpatrick and Keller, 2015), supported by gene annotation (see details in method sections). Once candidate adaptive loci have been identified, it is possible to assess genomic offset/genomic vulnerability that measures the change of the adaptive genetic composition in need to track the future climate shifts (Fitzpatrick and Keller, 2015; Bay et al., 2018; Rellstab et al., 2021). Furthermore, recent landmark studies have incorporated migration and dispersal into assessment of genomic vulnerability (Gougherty et al., 2021; Sang et al., 2022), which provide critical and promising information to guide adaptive management interventions for species keeping pace with future climate change, but have not been tested in many cases.

**FIGURE 1:**
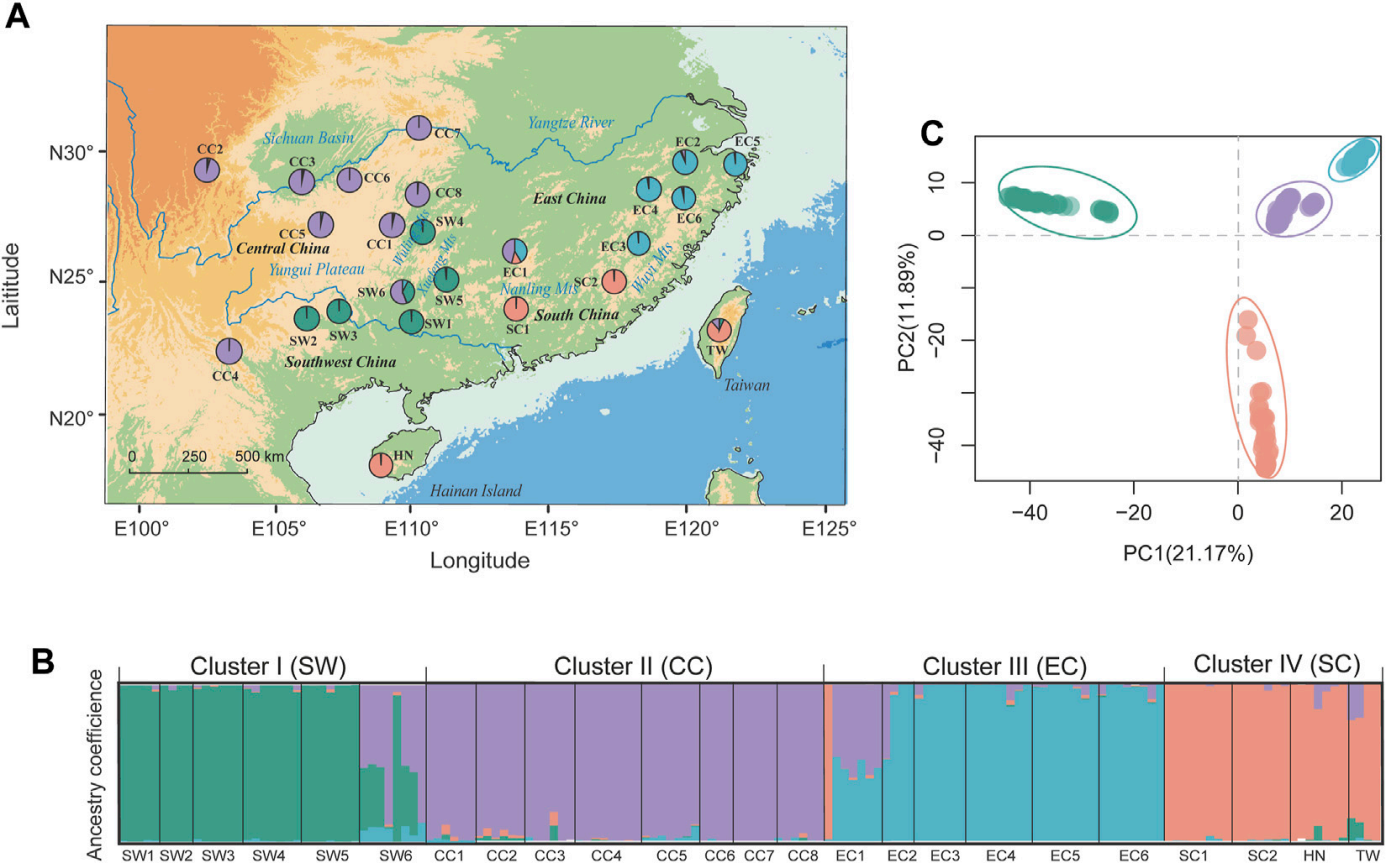
Genetic population structure of *T. hemsleyanum* as revealed by STRUCTURE (left panels) and DAPC analysis (top-right panel). **(A)** Geographic distribution of 24 populations with pie charts representing the ancestral components inferred by STRUCTURE analysis at *K* = 4. **(B)** Histogram of population assignment using STRUCTURE analysis with *K* = 4. The colored segment in each bar represents the individual ancestry coefficient of inferred clusters (or “gene pool”). **(C)** Population assignment results inferred from DAPC (using the R package adegenet).

Here, we applied the above analytical methods to *T. hemsleyanum* (Vitaceae), one of the widespread components of China’s warm-temperate evergreen forest. This diploid, perennial herb is endemic to subtropical China (also occurs in southern Hainan and Taiwan Islands) with a distribution that spans 18° of longitude, 13° of latitude and 1,000 m of elevation (Figure 1), which makes it an ideal model species to investigate variation in local adaptation to climate. In previous studies, substantial phenotypic and genetic variations were observed within natural populations of this species. Population germplasms of different geographic origin vary substantially in morpho-agronomic traits (Zhu et al., 2015; Yang et al., 2019) as well as in phytochemicals and pharmacological activities (Jiang, 2015; Yin et al., 2021). Regarding the genetic structure, our previous study indicated that *T. hemsleyanum* populations were clustered into four genetically distinct groups (Wang et al., 2015), inhabiting different floristic regions of subtropical China that vary in climatic conditions (Wu and Wu, 1998; Wu et al., 2010). This result points to the potential importance of climatic difference among flora in shaping patterns of intraspecific genetic variation. However, the amount of genomic variation determined by climate factors, the key climatic drivers, and the possible genetic targets (SNPs and genes) remain unknown. Beyond that, *T. hemsleyanum* is also an endangered medicinal plant in the official protection list of crop germplasm resources (Hu et al., 2021). In recent decades, *T. hemsleyanum* experienced substantial natural population declines as a result of human over-exploitation coupled with its specific climatic requirement for growth (Wang et al., 2018; Guo et al., 2019), which will undoubtedly increase the risk of maladaptation of local populations in face of future climate changes of subtropical China. As forecasted by robust climate models, subtropical China will be subjected to a rise in temperature, an enhancement of spatial heterogeneity in precipitation and an enlargement of arid regions by the end of the century (TCNARCC, 2011; Zhao and Wu, 2014). However, the risks that this climate sensitive species may face from these future climate challenges and where it is most effective in achieving conservation goals are still unknown.

Here, we sampled twenty-four populations of *T. hemsleyanum* across subtropical China and surveyed their genomic variation using specific-locus amplified fragment sequencing (SLAF, Sun et al., 2013). Our main objectives were to 1) evaluate the contributions of climate and geography to explaining patterns of genomic variation and characterize the key climate variables with the largest influence, 2) identify signatures of climate adaptation (i.e., via OA, EAA and annotation), and explore how locally divergent selection shaped patterns of allele frequencies changes of candidate adaptive SNP loci across species range, and 3) quantify and map which populations might be vulnerable to future climate change. Our study will facilitate a better understanding of the adaptation basis of China’s warm evergreen forest species and contribute to genetically informed measures for *T. hemsleyanum* resources management under shifting climates.

## 2 Materials and methods

### 2.1 Plant materials and genotyping

In this study, we collected fresh foliar samples of 252 georeferenced individuals at 24 independent natural sites (>50 km separation) (Supplementary Table S1; Figure 1). We chose 156 individuals for sequencing, including 132 individuals screened for variation previously using cpDNA and microsatellite loci (Wang et al., 2015), and 24 newly collected samples from Guangxi and Taiwan Island (Figure 1). Sampling locations were chosen to 1) cover entire species geographic and climatic distribution in mainland China and southern islands, including South/East China (SC1, SC2; EC1–EC6), Central China (CC1–CC8), Southwest China (SW1–SW6), Hainan (HN) and Taiwan (TW) Island; 2) represent major intraspecific phylogeographic clades identified in the previous study (Wang et al., 2015).

Total genomic DNA was isolated from the silica gel dried leaf material using Plantzol Reagent (Invitrogen). SLAF sequencing libraries were prepared using the HaeIII and RsaI restriction enzymes. The resulting 314 to 414 bp fragments (with barcodes and dual-index sequencing adaptors) were excised and purified with the Qiagen Gel Extraction Kit. After the library quality inspection, paired-end sequencing (the read lengths were 126 bp) was conducted on an Illumina HiSeq 2500 platform (Illumina, Inc., San Diego, CA, United States).

After trimming sequencing adapters, the raw reads were filtered for low-quality bases with a quality score less than Q20. Trimmed reads were demultiplexed using the Illumina bcl2fastq2 conversion software (Illumina, CA, USA). We aligned the reads to the *T. voinierianum* reference genome (Cai et al., 2021) using BWA-MEM (Li, 2013). *T. voinierianum* is a close relative to *T. hemsleyanum* with complete genome sequences available. SNP calling for each aligned sample were performed using GATK v.3.3 (DePristo et al., 2011) with HaplotypeCaller and GenotypeGVCFs tools. The SNPs identified by both methods were retained. Further SNP filters for subsequent analysis were applied with VCFtools v.0.1.14 (Danecek et al., 2011) and the “populations” program implemented in STACKS v.1.27 (Catchen et al., 2011) with the following criteria: a Phred pass score of 30, a mean maximum depth per locus of 100× across all sites (to avoid SNPs from over-represented organelle reads or falsely aligned paralogs), and a minimum read depth threshold of 6× for each sample. Furthermore, the minor allele frequency (MAF) per locus has to be greater than 5%, and only bi-allelic type of SNPs informative in at least 75% of the samples and 70% of the sites were retained. To reduce linkage, SNPs in strong linkage disequilibrium were removed using PLINK v.1.9 (Purcell et al., 2007), in a window size of 100 bp and a window step of 10 bp and a pairwise genotype correlation *r* ^*2*^ > 0.5.

### 2.2 Environmental data

We retrieved 35 bioclimatic variables (see Supplementary Table S2) for each sampling site from the WorldClim v.1.4 (Hijmans et al., 2005) and one derived set ENVIREM (Title and Bemmels, 2018). WorldClim variables (BIO1–BIO19) are generated from interpolation of mean monthly weather station climate data from 1960 to 1990 (centered on ∼1975). ENVIREM includes an expanded set of 16 bioclimatic variables to complement the WorldClim dataset, most of which are relevant to the physiological and ecological processes of plants (Title and Bemmels, 2018). For the future predictions, this study used statistically downscaled, bias-corrected CMIP6 general circulation models (GCMs) with high resolution. To account for uncertainty in model projections, a composite average of six GCM models (MRI-ESM2-0, BCC-CSM2-MR, IPSL-CM6A-LR, CanESM5, CNRM-ESM2-1 and MIROC-ES2L) with low amount of interdependence (Brunner et al., 2020), was developed for the time period 2061–2080 (centered on 2070), and for two shared socioeconomic pathways (SSPs)—SSP370 and SSP580. The raster files for future data downloaded from WorldClim were further utilized to generate ENVIREM variables for all GCMs and the SSP scenario using online pipelines (http://envirem.github.io/ENVIREM_tutorial.html). All variables for downstream analysis in this study were obtained at 30-s resolution (∼1 km^2^), and were scaled, centered, log-transformed (if appropriate) and tested for normality. To reduce multicollinearity, climatic cells with sampling records from all 35 variables were checked for Spearman’s correlation coefficient in R. Ultimately, a subset of 11 variables (see Supplementary Table S2) that lacked strong correlation (|*r*| < 0.75) were considered for environmental association analysis.

### 2.3 Genetic diversity and population genetic structure

Population genetic diversity parameters, including expected heterozygosity (*H*_E_), percentage of polymorphic loci (*P*) and Wright’s inbreeding coefficient (*F*_IS_) per population were estimated using the “populations” module of STACKS v.1.27 (Catchen et al., 2011). We utilized an analysis of molecular variance (AMOVA) to evaluate the genetic divergence among populations and geographic regions in Arlequin v.3.5 (Excoffier and Lischer, 2010), and the significance of *F*_ST_ values were assessed with 1,000 permutations. We investigated population genetic structure using STRUCTURE v.2.3.4 (Pritchard et al., 2009). For each number of cluster (*K*) that varies from 2 to 10, we run 10 repetitions with a 10,000 burn-in steps of and 1,00,000 Markov chain Monte Carlo (MCMC) replications. The program was utilized applying independent allele frequencies and an admixture model. The most probable values of *K* were determined by ∆*K* method (Evanno et al., 2005) using HARVEST software (Earl and vonHoldt, 2012). In addition, we also performed a discriminant analysis of principal components (DAPC) to infer the number of genetic clusters in the R package adegenet (Jombart, 2008; Jombart and Ahmed, 2011).

### 2.4 Assessing the role of climate and geography

We estimated the amount of genomic variation attributable to climate and geography by two different variance partitioning approaches—redundancy analysis (RDA) and generalized dissimilarity modelling (GDM). All climate variables were scaled and centered to account for difference in magnitude when calculating environmental distance. To test for the presence of regional climatic differences, the difference of each climate variable among the four genetic clusters (identified in STRUCTURE) were assessed using analysis of variance (ANOVA), followed by *post hoc* Tukey’s test. As a form of constrained ordination, RDA is applicable to genomic data (Forester et al., 2018) and permits to evaluate the total explanatory power of climate and geographical variables for genotypes (Legendre and Legendre, 2012). Using partial RDA, it enables the estimation of SNP variation independently attributed to geography, climate and their colinear portion (climate + geography) (Gibson and Moyle, 2020; Chang et al., 2022). We utilized the R package VEGAN to perform RDAs using climate variables and spatial variables (distance-based Moran eigenvector maps, dbMEM) as independent predictors (Oksanen et al., 2019). We generated dbMEMs based on geographic coordinates of sites using the *quickMEM* function in the ADESPATIAL package detailed in Borcard et al. (2018). Forward selection implemented in this function was performed to select significant dbMEMs. These dbMEM were finally included as predictors in our RDA models, which generate canonical axes representing the spatially structured genetic variation across species’ distribution range. The bioclimate variables, retained by Pearson correlation analysis, were further tested by forward selection implemented in the R package ADESPATIAL to find variables predictive for partitioning. Forward selection was applied at the *α* = 0.05 level and a maximum global adj*R* ^*2*^ threshold equal to the adjusted *R* ^*2*^ of the RDA model including all initial variables (in this case adj*R* ^*2*^ <0.318) with 10,000 permutations. In partial RDA, variance partitioning was carried out using population allele frequencies as the response variable in the two partial models that utilized the selected bioclimate variables as explanatory variables and the dbMEMs as conditioning variables and vice versa.

We additionally performed GDM to estimate the total and independent effects of environmental dissimilarity and geographic distance on genomic divergence. While GDM is in principle similar to RDA, it is based on a different statistical method, which employs non-linear regression to estimate the amount of population pairwise genetic distance attributed to pairwise differences of climate and dbMEM variables (Fitzpatrick and Keller, 2015). First, based on the same genomic data for variance partitioning in RDA, a population pairwise *F*_ST_ matrix was generated using the R package hierfstat (Goudet, 2005). Second, a site-by-environment predictor matrix was created including population IDs, geographic coordinates and the set of climate variables. Then, we fit GDM models in R package GDM using the matrix of genetic distance as the response variable and the climate variables as explanatory variables. We test the significance of each predictor variable by randomization tests (Shryock et al., 2021), and only included the predictor variables that significantly increase the explained deviance in the final fitted model (Manion et al., 2017). The relative importance of climatic predictors with regard to allelic turnover was estimated as the sum of I-spline basis functions (maximum height of each response curve; Fitzpatrick and Keller, 2015). To test the model robustness, we simulated 1,000 replicates, leaving out a random 10% of sites we sampled, and then performed GDMs the same before at each replicate (Murray et al., 2019).

### 2.5 Outlier and environmental association analysis

To identify genomic signatures of selection, we applied a combined analysis approach integrating three OAs (BAYESCAN, SELESTIM and BAYENV *X* ^*T*^ *X*) and EAA (BayPass). The three OAs we adopted are based on different demographic assumptions. BAYESCAN v. 2.1 (Foll and Gaggiotti, 2008) and SELESTIM v.1.1.4 (Vitalis et al., 2014) both used simple island model with migration. Specifically, BAYESCAN firstly generates population-specific and locus-specific *F*_ST_ coefficients while accounting for sample size variation, and then calculates the posterior probabilities of models with or without selection for each locus (Foll and Gaggiotti, 2008). In this study, three independent BAYESCAN runs were performed with prior odds of 10,000 following Lotterhos and Whitlock (2015), with 20 pilot runs of length 5,00,000, a thinning interval of 10, and a burn-in of 50,000. SNPs have consistent low false discovery rate (FDR < 0.05) in all three runs were considered as outliers. SELESTIM assumed that the study system followed a multinomial Dirichlet distribution of allele frequencies between populations as BAYESCAN, but relies on allelic frequencies instead of *F*_ST_ to identify loci under strong selective pressures (Hoban et al., 2016). This method uses Kullback-Leibler divergence (KLD) to estimate the distance of locus-specific coefficients of selection from the genome-wide effect of selection (Vitalis et al., 2014). The KLD was calibrated by simulated observed data (Vitalis et al., 2014). We carried 50 pilot runs with length of 5,000 to tune the model parameters, which is followed by 10,00,000 iterations with a burn-in of 1,00,000 and a thinning interval of 20. The threshold value of the empirical distribution of the KLD based on a pod analysis was set to 0.01.

To account for more complex demographic histories, we performed a third outliers test using BAYENV *X* ^*T*^ *X* (Günther and Coop, 2013). This method uses a population covariance matrix to accounts for neutral genetic structure. The matrix was created using SNPs not significant in other OAs. To minimize the stochasticity in null model estimation, the matrix was generated from the mean covariance matrix across three replicated runs produced from 5,00,000 iterations each. We then perform three runs for 5,00,000 iterations with the covariance matrix, and calculate *X* ^*T*^ *X* across replicates for each SNP. SNPs with top 5% of ranked *X* ^*T*^ *X* values were deemed to be outliers.

To complement the OA method, we used BayPass v2.1 (Gautier, 2015) to detect signals of selection based on associations between allele frequencies and climate variables, while accounting for hierarchical structure of populations. BayPass is an elaboration on the model of BayEnv2 and is wrapped under the script Baypass_workflow.R implemented in pyRona v.0.3.7 (Pina-Martins et al., 2017). Prior to runs, a covariance matrix was generated with the full SNP data set using the similar algorithm implemented in BayEnv2. Baypass was run under a standard covariance model (STD) with default parameter settings. Significant associations were defined as having an eBPis greater than 3 and a Bayes Factor greater than 15 dB, which are thresholds consistent with other studies (Ahrens et al., 2019; Seabra et al., 2021).

### 2.6 Landscape modelling

We test for spatially explicit selection processes for independent candidate SNPs using the package GDM in R (Ferrier et al., 2007; Manion et al., 2017). We adopted a “single-SNP” approach following Dudaniec et al. (2018) and modelled each candidate SNP independently, regardless of genomic contexts. The complete set of candidate loci identified by OA (by at least two methods) and EAA methods were used for GDM since each of the method adopt a statistic approach that is uniquely valid to reveal signatures of selection, and there is only minor overlap in the number and identity of retained SNPs across approaches.

Given the strong interspecific genetic differentiation and significant correlation between genetic structure and environmental gradients in *T. hemsleyanum* (see results), false positives were expected for the OA and EAA methods even with a correction for population structure. As a result, we conducted two additional steps to control for false positives by GDM. Firstly, we randomly choose 200 SNPs from the full set of 30,252 SNPs, and incorporate the random sample as a “reference” in the GDM to investigate if explanatory power of a candidate SNP is higher than that of the reference group. Secondly, we integrated geographic distance in the GDMs to reveal if spatial isolation better explained the observed partial allelic turnover across climatic gradients. This acts as a second screening for loci that predominantly respond to neutral genetic processes (i.e., isolation by distance). Overall, we tried to reduce false-positive rates, 1) by comparing the response of SNP-specific partial allelic turnover to climate variables with the response to geographic distance and 2) by comparing the response of each candidate SNP with reference SNP group to test whether a given locus explained a higher deviance within the GDM than a random sample of genetic variation.

We created the population pairwise *F*_ST_ matrices using the package HIERFSTAT in R (Goudet, 2005) and applied GDM to each of the candidate SNPs identified by EAA and OA. To evaluate the role of a specific SNP in selection processes, we ranked the SNP-specific allelic turnover functions in relation to each climate variable in two different ways as described in Dudaniec et al. (2018). Within each SNP, the ranking per variable is based on the partial allelic turnover magnitude relative to the other variables included in the GDM model. Across SNPs, the ranking per SNP is based on the explained deviance ranked relative to full candidate data set. After filtering of SNPs that 1) had the highest magnitude of partial allelic turnovers related to one of the climate variables rather than geographic distance and 2) explained higher model deviance than the reference SNP group (which had an explanatory power of 10.05%), 275 of 497 previous identified SNPs were retained.

### 2.7 Genomic contexts of candidate SNPs

The positions of candidate SNPs accompanying coding genes were identified based on gene models on the *T. voinierianum* genome (Cai et al., 2021) using BEDTools (Quinlan and Hall, 2010) and SnpEff (Cingolani et al., 2012). The gene models were predicted based on the *T. voinierianum* transcriptome (Matasci et al., 2014) and *Vitis vinifera* proteomes as described in Cai et al. (2021). The genes that are 1,000 bp upstream or downstream from the candidate SNPs were also presented (if any) to catch possible regulatory sites located in intergenic regions (Harris and Nielsen, 2014; Ahrens et al., 2019).

### 2.8 Genomic offset under future climates

For this threatened and endangered species, we are particularly interested in identifying spatial regions or populations at highest risk of future maladaptation, and where migration will be most effective to maintain the current status of adaptation. To do so, we extended the GDMs to estimate genomic offset, also called genomic vulnerability (Bay et al., 2018), which represents the disruptive effect of future climate change on contemporary genotype-climate associations (Rellstab, 2021). In this study, we followed a novel developed method in Gougherty et al. (2021) that incorporate migration into predict genomic models and used GDMs to calculated three metrics of genomic offsets that termed local, forward and reverse offsets. We fit GDMs to *F*_ST_ of the 275 retained candidate SNPs under putative selection. The fitted models were predicted to climate in 2070 and two shared socioeconomic pathways (SSPs)—SSP370 and SSP580. As described in Fitzpatrick and Keller (2015), local (classic) offset was calculated by estimating *in situ* allele frequencies shift at the robust set of climate-adaptive loci that a resident population required to response to local climate changes in 2070 (assuming no migration). Forward and reverse offsets were recently developed by Gougherty et al. (2021). For forward offset estimates, we firstly calculated the genomic offset of each current grid cell within the extant range of *T. hemsleyanum* to all future grid cells within China. Then we identified minimum offset among genomic offsets estimated across all future grid cells (termed “forward offset”), which assumes unconstrained dispersal of *T. hemsleyanum* populations to any location within China. High values of forward offset indicate a low adaptation capacity of the population to all future climates of China and a high chance of extinction in current genotype–climate relationships across the landscape. Besides, the distance and initial bearing that populations would migrate/disperse to the grid cell that minimized forward offset were estimated in R package geosphere (Hijmans et al., 2017) using the *distGeo* and *bearing* functions, respectively. Reverse offset is predicted by identifying the minimum offset among populations under current climate and “hypothetical” populations in 2017 and, both within the current range of this species. High reverse offset indicates the future novelty of genotype-climate relationships, since such relationships are not existed at any location of current landscape. The Spearman’s correlations between population structure, genomic offsets and climate variable shifts were quantified in R. To visualize genomic vulnerability to climate change, we presented an RGB image using ArcGIS 10.1 to simultaneously map all three genomic offset measures as red, green and blue bands, respectively, in geographic space for the year 2070. Prior to plotting, the values of each color band were rescaled to their quantiles (analogous to a histogram equalization) to illustrate the full range of each band.

## 3 Results

### 3.1 SLAF-seq and SNP calling

In this study, a total of *c.* 850 million reads was generated from all individuals with an average Q30 of 95.69% and a GC content of approximately 41.33% (Supplementary Table S3). The average number of reads per sample is 5.45 × 10^6^ (minimum: 2.02 × 10^6^; maximum: 13.17 × 106). High-quality SLAF tags (68,13,573 in total) were identified throughout the genome with an average of 12.05-fold sequencing depth (Supplementary Table S3). Subsequently, a total of 1,28,15,312 SNPs in 16,86,020 polymorphic SLAF tags were obtained. Of the 156 samples, 78.2% had a mean depth greater than 10×. After filtering for read depth and missing data (as described in the Methods), we retained a total of 30,252 high quality SNPs across samples.

### 3.2 Genetic diversity and population structure

Based on the high-quality SNP data, the genetic diversity of 24 populations in *T. hemsleyanum* were calculated and summarized in Supplementary Table S1. Nei’s genetic diversity (*H*_E_) was similar across populations (mean *H*_E_ = 0.364, SD = 0.025) and varied from 0.045 (SW1) to 0.125 (EC2). The *F*_IS_ values ranged from −0.044 (EC6) to 0.098 (EC1) with a mean value of 0.038. Significant pairwise genetic differentiation was detected for all population pairs (mean *F*_ST_ = 0.289, *p* < .001), ranging from 0.079 (between CC3 and CC6) to 0.455 (between EC2 and SW1) (Supplementary Table S4). Bayesian clustering implemented in STRUCTURE supported an optimal clustering at *K* = 4 (Supplementary Figure S1). The geographical assignment pattern of the individuals in the four clusters were almost concordant with to the geographical regions of Southwest [“*SW*” (green; pops SW1–5)], Central [“*CC*” (purple; CC1–8)], South [“*SC*” (tangerine; SC1–2, and adjacent island populations HN, TW) and East China [“*EC*” (light blue; EC1–5)] (Figures 1A, B). We observed high probabilities of ancestry to a given cluster for all populations, except that two populations (i.e., EC1, SW6) exhibited homogeneous levels of admixture. DAPC revealed a grouping pattern in concordance with that observed from STRUCTURE analysis, and the first two PCs explained 21.17% and 11.89% of the genetic variation, respectively (Figure 1C). AMOVA indicated that 37.01% of the genomic variation was distributed among regional groups (SW, CC, SC and EC) (*F*_CT_ = 0.37, *p* < 0.001), while 39.25% of the variation occurred within populations of *T. hemsleyanum* (Supplementary Table S5).

### 3.3 Impacts of climate and spatial variables on genome-wide population differentiation

#### 3.3.1 RDA analysis

**FIGURE 2:**
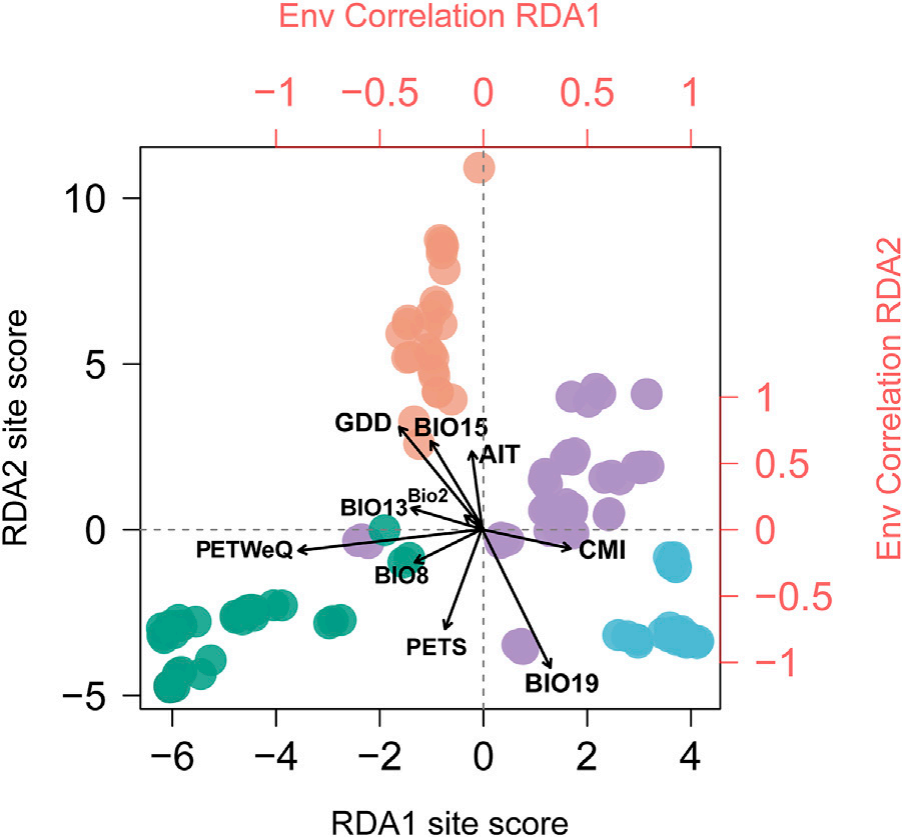
Biplot of Redundancy analysis conditioned on geography. Individuals are colored in corresponding with genetic clusters inferred by STRUCTURE analysis (see Figure 1). Top and right axes in red displayed the correlation of each climate variable (see Supplementary Table S2 for variable abbreviations) with RDA1 and RDA2 axes, respectively. Black vectors represent climate variables.

We performed RDA using partially constrained ordination (without accounting for spatial structure), whereby ten climate variables were identified as significant predictive of genetic variation among populations (Table 1), with one factor (PETS: Monthly variability in potential evapotranspiration) excluded. The most predictive variables include BIO15 (Precipitation Seasonality), GDD0 (degree days above 0°C) and PETWeQ (Mean monthly PET of wettest quarter). In Partial redundancy analysis (*p*RDA), four dbMEM were retained as sufficient to explain the geographic structure among populations (Supplementary Table S6; Supplementary Figure S2). Among these four axes, dbMEM2 and dbMEM4 described broad-scale spatial structure, whereas dbMEM5 and dbMEM6 described fine-scale structure (Supplementary Table S6; Supplementary Figure S2). dbMEM2 contributed most variation of any single spatial variable (6.19%; Supplementary Table S6). Cumulatively more variation was explained by broad-scale spatial variables (8.30%) than fine-scale ones (5.95%). Forward selection constrained on dbMEMs retained ten climate variables (Table 1; Figure 2), which is predictive while accounting for spatial effect. The strongest predictor contributing to the total variation was BIO19 (Precipitation of Coldest Quarter; 11.72%), followed by PETWeQ (3.29%) and GDD0 (3.27%), while AIT (Aridity Index Thornthwaite) was the weakest predictor (Table 1; Figure 2).

**TABLE 1:** Climate variables retained by forward selection and their contribution to genomic SNP variation using RDAs. The climate variable definitions are in Supplementary Material.

RDA (not constrained on space)			Partial RDA (constrained on space)		
Variable	Contribution to RDA model (*R* ^2^.adj%)	*p*	Variable	Contribution to RDA model (*R* ^2^.adj%)	*p*
BIO15	6.38	0.000***	BIO19	11.72	0.002**
GDD0	5.95	0.000***	PETWeQ	3.29	0.002**
PETWeQ	5.66	0.000***	GDD0	3.27	0.002**
PETS	4.3	0.000***	PETS	3.27	0.038*
BIO19	1.92	0.000***	BIO15	2.54	0.002**
BIO13	1.79	0.000***	BIO2	1.67	0.002**
BIO2	1.28	0.000***	CMI	0.85	0.002**
PETWaQ	1.11	0.000***	BIO8	0.67	0.002**
AIT	0.77	0.004**	BIO13	0.56	0.002**
BIO8	0.75	0.004**	AIT	0.25	0.002**

**FIGURE 3:**
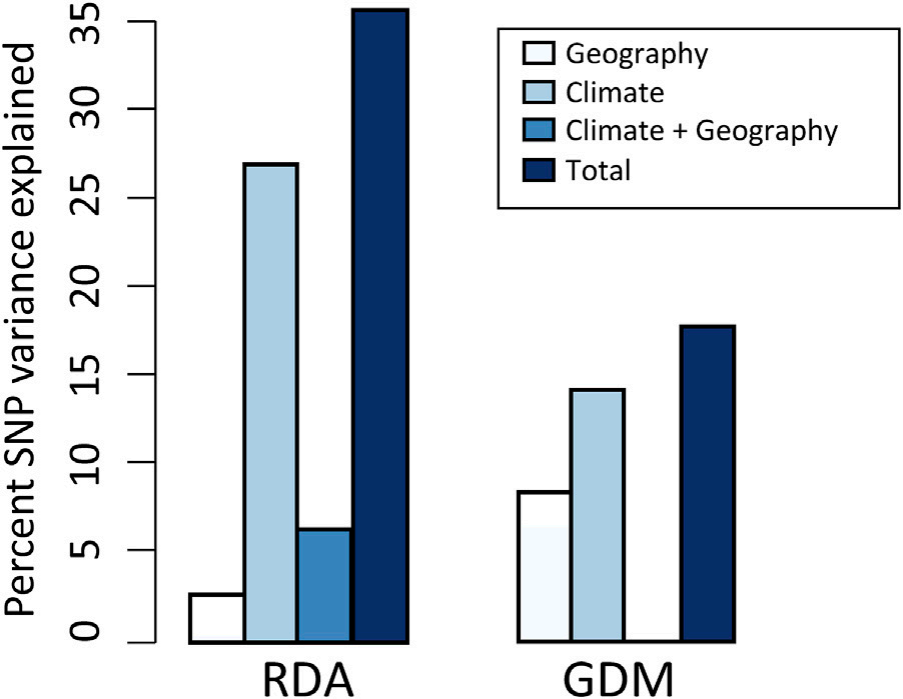
Results of variance partitioning estimated by RDA and GDM models. The total in legend means the genetic variation that climate and geography jointly explained, and the climate + geography means the collinear part of genetic variation. Note that GDM cannot estimate the contribution of climate + geography.

Using RDA, the climate (ten variables identified by forward selection) and geography both explained significant proportions of genetic variation (11.9%–31.8%, *p* < 0.001; “combined fractions” in Table 2). To further decompose their contribution, we performed partial RDA, which revealed that climate and geography jointly explained 36.4% of the inter-population genetic variation (“total explained,” Table 2). A minority of this (6.4%) was attributed to the collinear portion of climate and geography (Figure 3), that could represent the effects of clinical climate factors. Considering their independent effects, climate alone explained a substantially larger fraction of genetic variation than the geography alone (27.5% vs. 2.5%; Figure 3).

**TABLE 2:** RDAs to partition genomic variation among *T. hemsleyanum* populations into climate, geography and their combined fractions.

Combined fractions	*R* ^2^	*p* (>F)
F ∼ clim.	0.318	0.001***
F ∼ geo.	0.119	0.001***
Individual fractions		
F ∼ clim.|geo.	0.275	0.001***
F ∼ geo.|clim.	0.025	0.001***
F ∼ geo.+ clim.	0.064	—
Total explained	0.364	—
Total unexplained	0.636	—
Total	1	—

Notes: F, population allele frequencies matrix; RDA tests form, F ∼ independent matrices|covariate matrixes; clim., ten climate variables identified by forward selection; geo., four retained dbMEMs. Total explained: sum of adjusted *R* ^2^ of each fraction. The significance for confounded fractions (geo. + clim.) was not estimated. ***p < 0.001.

The correlations of climatic predictors with each RDA axes indicated that their contribution to population genetic variation varies geographically across the distribution range and therefore the climate variables acting as leading force in driving divergent selection regarding the geographic clusters being compared. Populations from South China (cluster IV) are exposed to greater growing degree days in comparison to populations from rest of the range; eastern coastal populations (cluster III) experience more winter precipitation; and southwestern populations (cluster I) experience highly potential evapotranspiration in warm and rainy seasons (Supplementary Figure S3). Moreover, we detected significant difference among the four genetic clusters that may contribute to divergent selection (*p* < 0.0001 for all tests; Supplementary Figure S3), indicating that these regional clusters tend to occupy habitats characterized by more or less unique climate features.

#### 3.3.2 GDM analysis

The full GDM (climate + geography) model explained 17.6% of the deviance in spatial patterns of genetic variation among populations (*p* < 0.001) (Figure 3; Supplementary Table S7). The models accounting for individual effect of geography and climate explained 8.1% and 14.3% of the deviance, respectively (Figure 3). As with RDA, this finding suggests that a larger proportion of variation is attributable to climate differentiation than to geographic isolation. In climate GDM model, the sums of I-spine basis functions (Supplementary Figure S4) indicated that BIO15 (Precipitation Seasonality) had the greatest magnitude in partial allelic turnover response (importance weight = 0.330), followed by BIO19 (Precipitation of Coldest Quarter; importance weight = 0.298). It is worth noticing that these two variables were consistently identified as significant predictors contributing to SNP variation in both GDM and RDA analysis (see Supplementary Tables S8, S9 for variable contributions in GDM models).

### 3.4 Identification of candidate adaptive SNP

**FIGURE 4:**
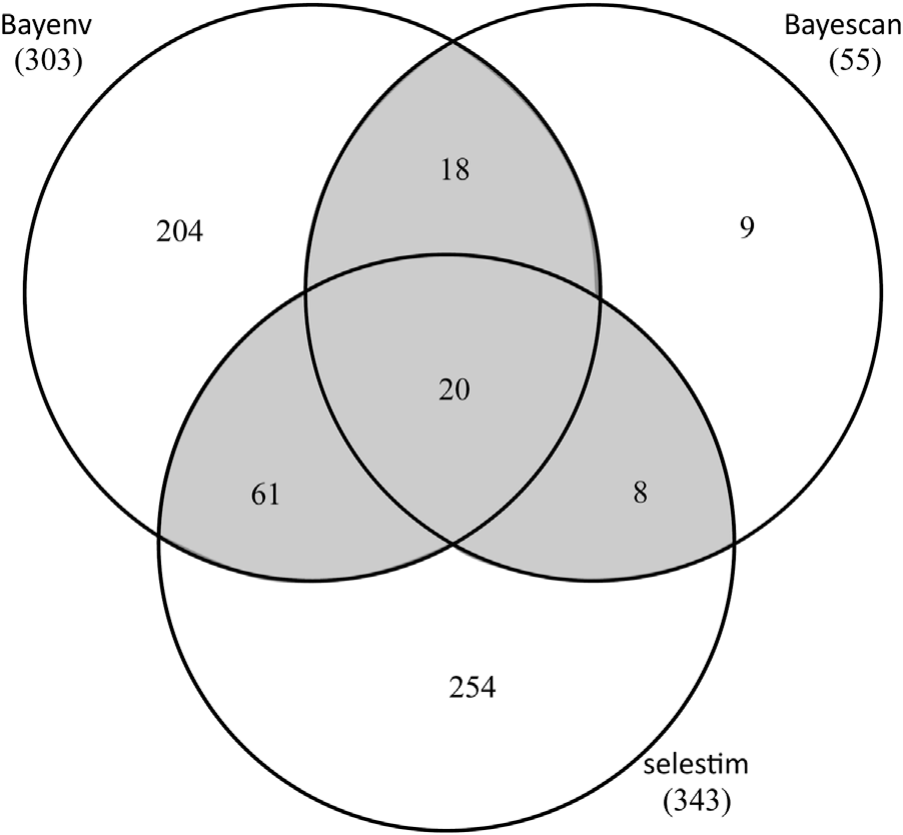
Number of *T. hemsleyanum* SNPs detected in three *F*_ST_ outlier tests. Shaded overlapping regions (outliers in >2 tests) represent SNPs deemed as outliers in this study.

By OA, a total of 107 SNPs was detected in at least two of the three tests (Figure 4; Supplementary Table S10). Specifically, BayeScan (see Supplementary Figure S5), BayENV *X* ^*T*^ *X* and Selestim identified 55, 303, and 343 putatively adaptive SNPs, respectively. 20 SNPs were identified in all three tests and 87 in two tests, with the rest SNPs exclusively to BayeScan (9), BayENV *X* ^*T*^ *X* (204) and Selestim (254) (Figure 4). Using EAA analysis (Baypass), 402 SNPs were identified with significant associations with climate variables, and 14 of these SNPs were related to multiple climate variables (Supplementary Table S11). Collectively, a total of 497 SNPs was identified to be outliers by multiple OA methods and/or to be associated with at least one of the eleven climate variables tested, representing 1.7% of the SNP dataset. Of these candidate SNPs, only 12 SNPs were shared between OA and EAA approaches (Supplementary Tables S10, S11).

GDMs were independently applied to the 497 candidate SNPs to quantify partial allelic turnovers through climatic gradients (Supplementary Table S12). For each variable, we presented GDM results for the top 150 SNPs with the greatest magnitude of partial allelic turnovers in Supplementary Figure S6, which revealed varying gradients and strengths of selection acting across a range of loci. For example, the SNPs associated with geography (Supplementary Figure S6A), BIO19 (Supplementary Figure S6F) and PET seasonality (Supplementary Figure S6J) mostly appeared to reach the highest allelic turnover magnitudes at positions where the greatest change occurs along environmental gradients, while SNPs associated with BIO15 mostly ceased allelic turnover beyond a precipitation seasonality cut-off of 58 (Supplementary Figure S6). On average, the magnitude in partial allelic turnovers per climate variable for the 497 SNPs were highest in relation to BIO19 (0.147), followed by BIO13 (0.141) and PET seasonality (0.120) (Supplementary Table S12). BIO13 (Supplementary Figure S6D) and GDD0 (Supplementary Figure S6I) drove the most variable magnitudes of partial allelic turnover, both of which had distinct thresholds of turnover recognizable for each related locus.

Among the 497 previous identified candidate SNPs, 6 SNPs (1.2%) showed non-significant allelic turnover response to any of the variables, and were not interpreted further. 50 of the 497 SNPs (10.1%) had less magnitude in partial allelic turnover associated with climate variables than with geographic distance (and considered to be possible false positives). The “reference SNP group” explained 10.5% of the deviance in GDM model, and therefore the SNPs with explanatory power less than 10.5% were also excluded. Finally, we retained 275 SNPs for further functional annotation.

### 3.5 Annotation

Of the 275 putative adaptive SNPs we identified, 219 occurred in SLAF scaffolds that aligned to contigs of *T*. *voinierianum* genome assembly, and reside in different genomic regions including coding, intron and intergenic regions. No candidate SNPs were found within regulatory regions. Of the 219 SNPs, 92 SNPs were predicted to fall within genes. 31 (33.7%) out of the genic SNPs were exonic variants, including two identified in OA, 28 in EAA test (associated with climate variables) and one in both tests (Table 3). The 31 SNPs uncovered gene functions associated with abiotic stimuli response, cell cycle progression, flora reproductive development and terpenoid synthesis. Full annotation results of all candidate SNPs are given in Supplementary Table S13.

**TABLE 3:** Gene annotation and associated variables for potentially adaptive SNPs. SNP IDs, gene function on the *T. voinierianum* genome are shown. The detection method (PD/EA tests) and partial allelic turnover by variables predicted by GDM are listed for each annotated locus. The SNP presented had 1) higher allelic turnover responses associated with climate variables relative to geographic distance, and 2) a higher explained deviance (%) in GDMs than the reference SNP group. The climate variable definitions are in Supplementary Material.

SNP_ID	Gene annotation from the *T. voinierianum* genome		Detect. Meth.	% GDM	Partiall allelic turnover by variable											
	Symbol	Description			GEO	BIO13	BIO15	BIO19	BIO2	BIO8	AIT	CMI	GDD0	PETs	PETWaq	PETWeQ
106296_81	RH7	DEAD-box ATP-dependent RNA helicase 7	Both	19.62	0.27	0.00	0.05	0.00	0.00	0.26	0.00	0.09	0.25	0.48	0.00	0.00
108307_57	APX1	L-ascorbate peroxidase 2, cytosolic	EAA	10.05	0.14	0.07	0.16	0.00	0.04	0.00	0.00	0.00	0.14	0.21	0.13	0.22
108813_20	GALT31A	Beta-1,6-galactosyltransferase GALT31A isoform X1	EAA	19.81	0.29	0.09	0.00	0.00	0.00	0.40	0.04	0.00	0.00	0.02	0.00	0.00
111595_169	SHT	Spermidine hydroxy-cinnamoyl transferase	EAA	28.44	0.18	0.09	0.00	0.36	0.57	0.00	0.01	0.00	0.04	0.44	0.08	0.09
112920_33	At4g11810	SPX domain-containing membrane protein	PD	31.13	0.08	0.00	0.01	0.64	0.00	0.07	0.13	0.02	0.87	0.00	0.04	0.00
		At4g22990														
149183_189	BAM1	Leucine-rich repeat receptor-like serine/threonine-protein kinase BAM1	EAA	12.26	0.18	0.23	0.21	0.00	0.09	0.31	0.19	0.00	0.00	0.18	0.00	0.05
150607_97	CYCA2-4	Cyclin-A2-4 isoform X2	EAA	15.91	0.00	0.19	0.00	0.56	0.04	0.00	0.00	0.00	0.00	0.23	0.04	0.11
157676_241	NORK	Nodulation receptor kinase	EAA	18.54	0.01	0.00	0.71	0.00	0.01	0.00	0.00	0.37	0.00	0.00	0.00	0.51
165239_220	MS5	Protein POLLENLESS 3	EAA	13.89	0.00	0.00	0.00	0.00	0.00	0.00	0.00	0.39	0.00	0.00	0.00	0.08
182454_174	ECR	Very-long-chain enoyl-CoA reductase isoform X1	PD	25.52	0.24	0.63	0.00	0.02	0.00	0.51	0.17	0.00	0.00	0.00	0.00	0.04
183110_19	ABCG10	ABC transporter G family member 10	EAA	25.31	0.20	0.36	0.02	0.00	0.39	0.05	0.00	0.00	0.34	0.00	0.00	0.00
200811_248	Os01g0253300	Importin subunit alpha-4	EAA	18.93	0.00	0.19	0.18	0.00	0.38	0.13	0.00	0.34	0.22	0.11	0.00	0.15
210328_156	TPS9	Terpene synthase 9	EAA	13.73	0.18	0.00	0.10	0.00	0.00	0.05	0.00	0.52	0.24	0.00	0.00	0.41
226772_121	MYB3R1	Transcriptional activator MYB	EAA	15.84	0.25	0.00	0.00	0.02	0.16	0.17	0.00	0.00	0.00	0.26	0.06	0.00
234472_247	BHLH93	Transcription factor bHLH93 isoform X2	EAA	15.40	0.00	0.06	0.18	0.02	0.04	0.01	0.00	0.00	0.00	0.26	0.00	0.00

259725_242	GRDP2	Glycine-rich domain-containing protein	EAA	12.38	0.22	0.12	0.00	0.00	0.00	0.00	0.00	0.00	0.00	0.24	0.00	0.00
259831_218	Stk24	Germinal center kinase 1 isoform X3	EAA	21.16	0.00	0.00	0.24	0.66	0.06	0.10	0.01	0.00	0.00	0.03	0.00	0.26
275220_193	BHLH60	Transcription factor BHLH60	EAA	11.70	0.00	0.00	0.12	0.00	0.00	0.08	0.00	0.00	0.00	0.04	0.00	0.04
43765_162	PER64	Peroxidase 64	EAA	28.09	0.00	0.08	0.17	0.03	0.13	0.00	0.00	0.00	0.60	0.00	0.02	0.33
47867_74	LMK1	Putative LRR receptor-like serine/threonine-protein kinase	EAA	16.46	0.15	0.00	0.15	0.30	0.02	0.00	0.14	0.00	0.31	0.06	0.01	0.21
50095_180	PCMP-H88	Pentatricopeptide repeat-containing protein At1g08070	EAA	29.45	0.00	0.00	0.00	0.10	0.00	0.16	0.00	0.05	0.88	0.34	0.02	0.07
53321_68	WRKY70	Probable WRKY transcription factor 70	EAA	10.59	0.00	0.00	0.10	0.00	0.07	0.39	0.20	0.12	0.00	0.17	0.00	0.06
56811_114	SecA	Protein translocase subunit	EAA	19.56	0.23	0.00	0.00	0.00	0.85	0.11	0.00	0.00	0.00	0.00	0.00	0.00
		SecA, chloroplastic														
61513_232	RE2	Uncharacterized protein	EAA	15.54	0.31	0.00	0.38	0.00	0.04	0.00	0.00	0.29	0.00	0.32	0.00	0.01
		LOC107261244														
64046_224	HMA5	Putative copper-transporting ATPase HMA5	EAA	13.18	0.25	0.00	0.00	0.00	0.16	0.00	0.00	0.00	0.00	0.29	0.00	0.16
64578_209	DDB_G0268948	Putative methyltransferase	EAA	12.42	0.00	0.00	0.00	0.04	0.00	0.10	0.18	0.00	0.07	0.13	0.29	0.00
67001_214	BGAL6	Unnamed protein product, partial	EAA	19.84	0.29	0.21	0.00	0.00	0.00	0.37	0.17	0.00	0.00	0.00	0.00	0.00
68729_42	PAP15	Hypothetical protein VITISV_037278	EAA	13.75	0.00	0.00	0.00	0.00	0.00	0.00	0.17	0.44	0.04	0.35	0.00	0.06
71182_245	APC1	Anaphase-promoting complex subunit 1 isoform X1	EAA	16.64	0.06	0.05	0.19	0.08	0.20	0.12	0.05	0.00	0.25	0.00	0.14	0.02
8850096_188	LPXA	Putative acyl-[acyl-carrier-protein]--UDP-N-acetylglucosamine O-acyltransferase, mitochondrial	EAA	18.51	0.18	0.00	0.04	0.95	0.07	0.00	0.00	0.00	0.23	0.17	0.08	0.00
92082_176	ABCC10	ATP-binding cassette transporter member	EAA	15.20	0.00	0.00	0.24	0.00	0.00	0.12	0.05	0.45	0.18	0.39	0.00	0.21

### 3.6 Genomic offset and migration to climate change

**FIGURE 5:**
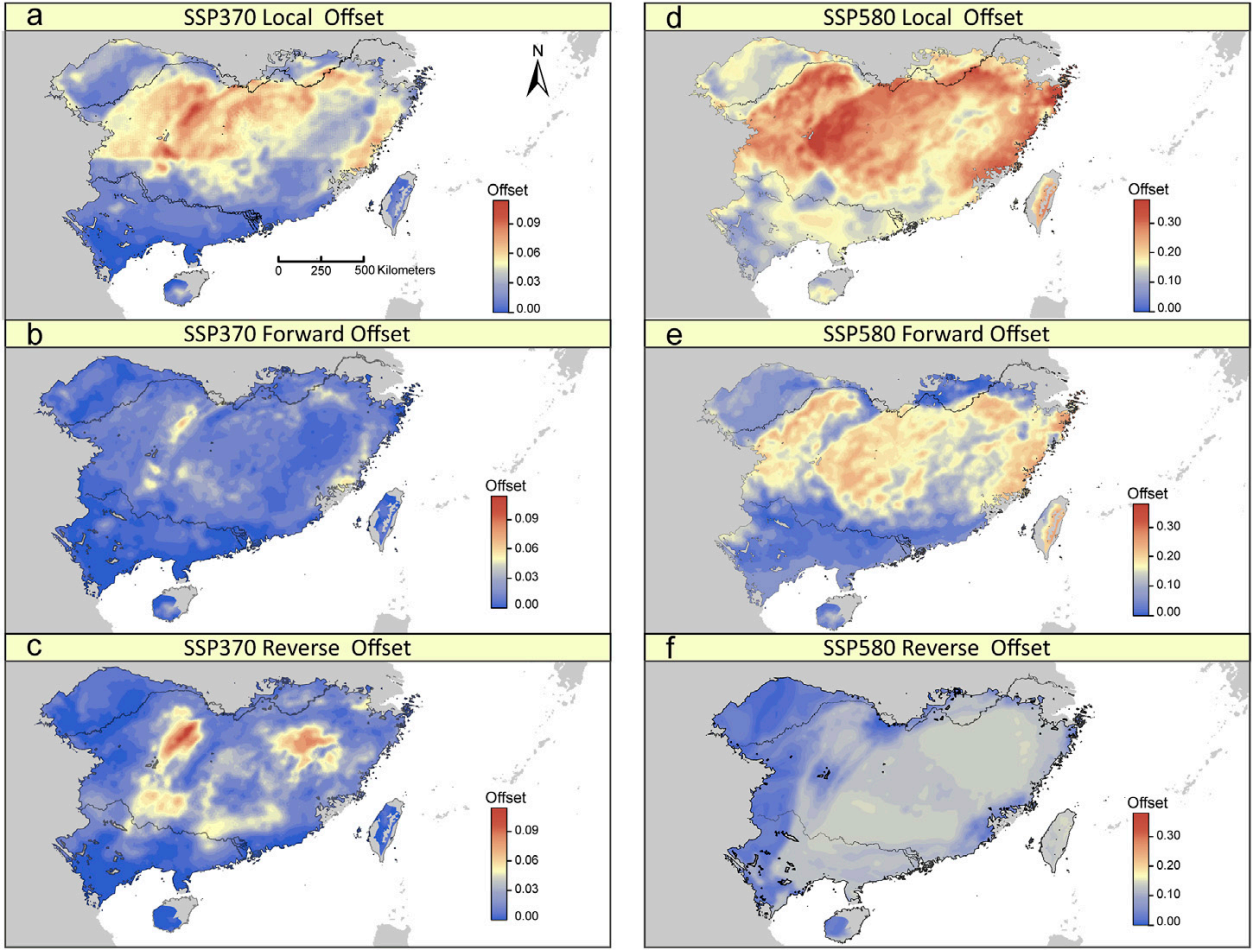
Spatial distribution of local, forward, and reverse genomic offsets estimated from GDM under SSP370 **(A–C)** and SSP580 **(D–F)** in 2070.

The GDM model based on 275 potentially adaptive loci explained 25.91% of the deviance. BIO19 was the most important predictor for the observed adaptive genetic variation (Supplementary Figure S7). Under a scenario of future climate change, although we found differing patterns of the local, forward and reverse offsets across the distribution range of *T. hemsleyanum*, some general patterns can be observed. The three genomic offsets were predicted be lowest for populations in the southern range margin of the mainland (Figure 5; Supplementary Figure S7). By contrast, the central-north part of the range, especially along eastern Yungui Plateau and Xuefeng Mts, have relatively high levels of genomic offsets (Figure 5; Supplementary Figure S7), indicative of relatively high future disruption of genotype-climate relationships in populations occupied current locations, and such effects of climate change cannot be mitigated by movement towards more suitable locations. Local and forward offsets were most strongly associated with shifts in BIO19, PET seasonality and arid index (Supplementary Figure S9). Besides, there is no obvious relationships between local/forward offset and population structure, except for a significant negative relationship existed between local offset and the estimated ancestry coefficients for the “South” cluster (Supplementary Figure S9).

**FIGURE 6:**
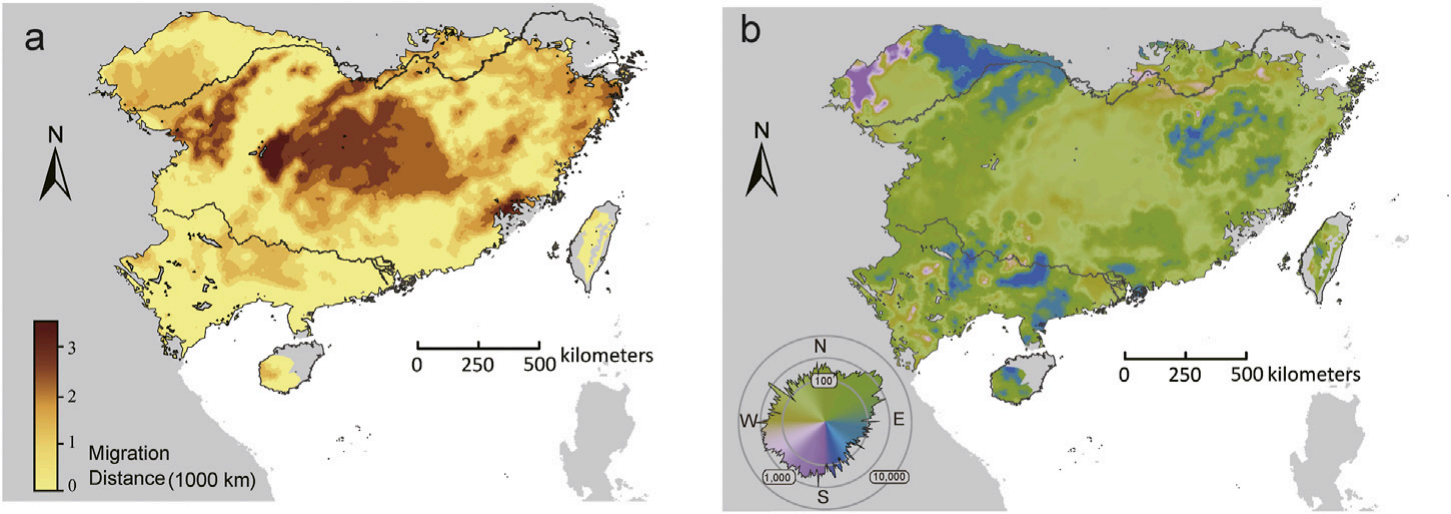
The initial bearing **(A)** and migrate distance **(B)** to locations that can minimize forward offset in the future climate scenarios (2070, SSP370). The polar histogram in **(B)** indicated the log_10_ number of cells within each bearing bin.

For the set of adaptive loci, few (<1%) locations had the distance to locations that minimized forward offset (*D*_min_) equals to zero. The longest *D*_min_ was predicted in central-north part of the range, whereas the shortest *D*_min_ mainly occurred in the southern and northwestern range edge (Figure 6A). Furthermore, the initial direction that populations would adhere to move to locations that minimize maladaptation risks varied throughout the range. The GDMs predicted an overall northward trajectory for most (71.14%) locations within the range, but substantial variations were observed beyond the core of the range, especially along the northwestern range edge, where populations showed eastward, westward, or even southward trajectories (Figure 6B).

## 4 Discussion

This study demonstrated a broad correlation between genetic and regional climatic difference in this species, and identified climate as a predominant force in driving current patterns of genetic structure. To trace footprints of climate-related adaptation, we adopted a landscape genomics framework to analyze population genomic data, from which candidate SNPs were discovered by OA and EAA methods, then further screened by SNP-specific GDM modelling and functional annotation. Based on the adaptive genomic variants, this study, for the first time, incorporated migration and dispersal into vulnerability assessment of this widespread species in subtropical China. We investigated most vulnerable populations that require proactive management efforts and candidate adaptive genes for breeding and other special agricultural aims. The results highlighted the role of heterogeneous climate conditions of subtropical China in shaping genomic structure and driving local adaptation among populations, facilitating future *T. hemsleyanum* conservation managements.

### 4.1 Regional climatic differences contribute to genomic divergence

In this study, we applied RDA and GDM to quantify the contributions of spatial isolation and climate variables to patterns of genomic variation, and obtained concordant results. Both methods identified a larger proportion of SNP variation attributed to climate than to geography (Figure 3), indicating that the assessed climate variables are more influential in driving the pattern of genetic variation in *T. hemsleyanum*, which has been frequently reported in plant species (Shryock et al., 2017; Vidaller et al., 2020; Feliciano et al., 2022). When we isolate the effect of climate variables, geography alone explained a rather small portion (RDA: 2.5%; GDM: 8.1%; Figure 3), which potentially reflect the absence of unmeasured spatially structured climate variables (Feliciano et al., 2022). Furthermore, our RDA and regional climatic differences revealed that climate predictors acting as key selective agents vary across specific geographic clusters (Figure 2; Supplementary Figure S3).

Unlike GDM analysis, RDA was able to quantify the contribution of the colinear fraction of climate and geography. This fraction, termed as induced spatial dependence (ISD), could represent impact of climatic gradients that are highly related to space (Borcard et al., 2011; Mikulyuk et al., 2011; Ma et al., 2020). While clinal gradients (e.g., latitudinal temperature gradients) are treated as key adaptive evolutionary forces in plant species (Adrion et al., 2015; Kooyers et al., 2015; Gibson and Moyle, 2020), the estimated influence of ISD on genomic variation of *T. hemsleyanum* was low (6.4%; Figure 3; Table 2). This eliminated some of the confounding effect of demography that could impact the inference of local adaptation.

Apart from quantifying the relative effects of climate and space, we also found specific climate factors that uniquely contributed to genomic variation. In subtropical areas, the survival and distribution of plants has been reported to be constrained by insufficient precipitation (Allan et al., 2010; Aguirre-Gutiérrez et al., 2019), and across the range of *T. hemsleyanum*, BIO19 and PETWeQ represented the most important predictors of genome-wide SNP variation when taking the effects of spatial isolation into account, which suggested that populations are responding to selective forces related to drought. This result agrees with the general consensus that *T. hemsleyanum* is best suited to moist hillsides or valleys and is a drought-sensitive species (Du et al., 2015; Song et al., 2017), and reinforces previous findings of distribution models that identified water availability as a major determinant of contemporary distribution range of this species (Wang et al., 2022). Beyond that, GDD0 was by far the most important variable in the temperature regime associated with genetic variation (Table 1), and its corresponding range was from 51,930 days degrees at Xingshan (CC7) in Central China to 1,03,932 days degrees at Janfengling (HN) in South China (Supplementary Table S1). The large variations in temperature conditions during growing seasons reflected differential heat requirement of *T. hemsleyanum* across regions, and this seemed to be a strong abiotic selective agent, especially between populations in southern and central regions. Indeed, local adaptation of populations for phenological traits in relation to accumulated heat conditions has been described in several species with wide distributions (Khoufi et al., 2013; Olson et al., 2013). As a result, we presume that similar pattern may also be found in *T. hemsleyanum* populations along the GDD gradient. Such hypothesis is testable in the future, with common garden experiments to assess phenological variation across natural populations that are predicted to differ at specific SNPs associated with growing degree days. This may also aid in the selection of populations genetically adapted in their phenology to face less GDDs and better adapted to cold stress.

Although in our study climatic selection processes appeared to be the most important in driving genomic variation, a large portion of the variation remains unexplained in RDA (63.6%) and GDM (77.6%) analysis, which accords with the other genetic (Jia et al., 2020; Boulanger et al., 2021) and community ecological studies (Ma et al., 2019) using similar approaches. This probably results from several factors not fully addressed in this study. Firstly, despite that many climate predictor variables were considered in this study, other ecological forces may also play a role; these may include biotic interactions, and abiotic factors that were unmeasured or occurred at relatively small scales (Geue et al., 2016; Gibson and Moyle, 2020). Secondly, the remaining variation can be attributed to balancing selection or neutral and/or stochastic process that maintain local allelic diversity, which may weaken the predictive power of the models. Thirdly, the four dbMEM variables generated here may not fully represent geographic heterogeneity present in subtropical China. Lastly, RDA analysis, which model linear associations between geography/climate and SNP loci, cannot fully capture non-linear statistical relationships. As a result, we also applied GDM to the SNP data and detected the non-linear SNP-climate relationships arise across the range of *T. hemsleyanum*; this would be critical to understand the process of local adaptation in the context of multivariate climates.

### 4.2 Detection of candidate SNPs by multiple methods

Combining population differentiation with EAA methods is a desirable way to identify potentially adaptive SNPs and reduce the rate of false positives (Martins et al., 2018; Lu et al., 2019). One notable aspect of these two methods employed in this study is the minimal overlap of SNPs we identified. Specifically, only 12 SNPs deemed as *F*_ST_ outliers were detected in BayPass, and between 2.70% and 9.67% of the genotype-environment associations were overlapped among two or more variables (Supplementary Tables S10, S11). The low levels of congruence are not surprising since the two methods captures different selection signatures (Eckert et al., 2010; Hancock et al., 2010; Martins et al., 2018; Lu et al., 2019). OA are generally more sensitive to detect strong signatures of divergent selection acting directly on new mutations, yet it is impossible to determine specific environment forces (de Villemereuil et al., 2014). EAA tests, on the other hand, performs better in identifying polygenic or weak selection signatures (Narum and Hess, 2011; de Villemereuil et al., 2014; Frichot and François, 2015) and are nowadays widely applied to explore adaptive loci with subtle variation across landscape (Jones et al., 2013; Martins et al., 2018). Concordantly, previous studies comparing OA and EAA have found little overlap in the significant SNPs between the approaches (Dudaniec et al., 2018; Lu et al., 2019). A caveat on using EAA is that a locus may not be significantly associated with environmental factors when it is advantageous across a range of environment conditions at the same time (Frichot et al., 2013). The GDM analysis included in our approach is complementary to EAA, since it is capable to simultaneously characterize relative allelic responses across predictor variables.

In forest species, climate adaptation is likely driven by polygenic alleles with small effects (Savolainen et al., 2013; Sang et al., 2022). In *T. hemsleyanum*, we surmise that a number of SNPs identified here are genetic variants of small effects according to previous reviews and case studies (Mackay et al., 2009; Rockman, 2012; Savolainen et al., 2013; Barghi et al., 2020). Indeed, climate explains a small to moderate portion of allelic turnover for majority of the SNPs detected by multiple models (Supplementary Figure S6; Supplementary Table S12), and the defined climate points of allelic turnover varies across adaptive variants (Supplementary Figure S6), suggesting that climate is affecting each SNP differently. This finding may reflect additive genetic variation related to many genes or genomic regions and multilocus patterns of adaptation (Shaw and Etterson, 2012). Furthermore, for the annotated candidates (listed in Table 3), especially for that highly supported by GDM, the observed *F*_ST_ changes (0.27–0.50) were not biased towards greater values (Supplementary Table S12), which indicated that a higher probability to identify a SNP under selection does not correlate with a greater shift of *F*_ST_ values across climatic gradients. Overall, these observations indicate that climate adaptation in *T. hemsleyanum* is polygenic and potentially related to both small- and large-effect genetic variants. Future experiments that investigate gene interactions may help to elaborate the polygenic basis of local adaptation (He et al., 2016; De Kort et al., 2022).

Detecting signals of local adaptation is complicated by the issue of disentangling geographically structured variation from adaptive variation (Hoban et al., 2016), which is especially relevant when environmental gradients are highly correlated with neutral structure (Lotterhos and Whitlock, 2015). Given the strong population differentiation and broad correlations between genetic clusters and floristic divisions (Supplementary Figure S10) in this species, we adopted multiple filtering and correcting steps in genome scans to better control for false positives. First, we selected only putatively adaptive SNPs found by two or three outlier tests (only diversifying selection) and excluded SNPs in relation to geography in EAA. Next, considering the small overlap of loci detected by OA and EAA methods, we used two subsequent screening approaches in GDM to control for false positives by 1) excluding loci that had an explanatory power not exceeding the “reference” SNP group and 2) excluding loci with highest partial allelic turnover response to geography. Finally, we focus our interpretation on SNPs located in stimuli response or other ecologically relevant genes, since they offer a better opportunity to elucidate gene function and climatic forces driving the current patterns of adaptive variation. Future whole genome sequencing of *T. hemsleyanum* individuals may facilitate more comprehensive investigation of genetic targets of selection and mining of adaptive loci valuable for breeding purposes.

### 4.3 Key genes with a local climate adaptation signature

We retained 275 SNPs with significant signatures of selection, some of which were located in exonic regions of genes associated with plant adaptation to abiotic environment (Table 3; Supplementary Figure S13). Of foremost interest are those genes that act as key regulators of abiotic stress tolerance. For example, one SNP 106296_81 (identified by both OA and EAA methods) resides in the *RH7* gene encoding a DEAD box helicase (Table 3). DEAD box helicases serve as important molecular tools in developing stress tolerant plants (Nidumukkala et al., 2019). In *Arabidopsis thaliana RH7* allelic mutants, plants showed developmental defects and high sensitivity to cold stress (Huang et al., 2016; Liu et al., 2016). We also found one SNP (108307_57) located in the *APX1*, which plays a vital role in adaptation of plants to a combination of drought and heat stress (Zandalinas et al., 2018). Not surprising that both EAA and GDM identified this SNP to be significantly associated with potential evapotranspiration (PET) (Table 3; Supplementary Figure S11), which is a climatic measure integrating temperature and humidity to reflect water availability (Rehana and Monish, 2021). We also identified one candidate SNP linked to a pentatricopeptide repeat (*PPR*) gene. In cotton, a single recessive mutation of a *PPR* gene reduces the heat accumulation (Kim et al., 2021). In this study, the SNP annotated to the *PPR* gene showed the highest magnitude of partial allelic turnover in response to GDD (0.88) and served as top-ranked SNPs for growing degree days (9) (Table 3; Supplementary Figure S12), suggesting that this *PPR* gene is among good candidates for further functional studies.

Beyond that, a role for gene regulation variation, not merely functional variation, for climate adaptation was indicated by the detection of genes encoding several families (*bHLH*, *MYB*, *WRKY*, etc.) of transcription factors (TFs), for example, *bHCH93*, identified as a key player in regulating flowering (Wang et al., 2017) and abiotic stress responses (Bhaskarla et al., 2020; Samarina et al., 2020; Wang et al., 2021a), and *MYB3R1* acting as a “master switch” in a variety of stress tolerance (Dai et al., 2007). The TF families and members we found here mostly match results in previous studies, such as bHLH transcription factors identified as outliers in *Corchorus olitorius* (Sarkar et al., 2019) and *Pinus taeda* (Lu et al., 2019), and MYB transcription factors that identified by both OA and EAA methods in *Brachypodium distachyon* (Dell’Acqua et al., 2014).

Furthermore, we note two candidate genes encoding A-type cyclin (*CYCA*) and anaphase-promoting complex (*APC*), both of which control cell cycle progression. Recent studies have begun to address the important role of cell cycle regulator, especially cyclins (such as *CYCA2;4* we identified here) and cyclin-dependent kinases (*CDK*s), in stimulus response of plants (Komaki and Sugimoto, 2012). *Arabidopsis thaliana* and maize were reported of cell cycle arrest and cell proliferation reduction in response to salt and drought stresses due to the disruption of cell cycle regulators (Kamal et al., 2021).

It is noteworthy that several genes involved in flora reproductive development and flowering time were also identified (Table 3). *GRDP2* encodes a novel glycine-rich domain protein that modulates flowering time and ovule development (Ortega-Amaro et al., 2015; Czolpinska and Rurek, 2018; Wang et al., 2021b), and was previously identified to be involved in environmental response in *Arabidopsis* (Mangeon et al., 2010; Ortega-Amaro et al., 2015). *BAM1* has been shown to regulate anther development in *Arabidopsis* (Hord et al., 2006), and *MS5* is known to be essential for male fertility in *Arabidopsis* and *Brassica* species (Glover et al., 1998; Zeng et al., 2021). The initiation of reproduction is an important transition of life cycle (Franks and Hoffmann, 2012). In plants, due to their sedentary life-style, the exact timing of flowering has strong impacts on reproductive success and, thus fitness (Anderson et al., 2011). As outlined in previous studies, selection can optimize flowering time to track suitable climatic conditions, thus contributing to plant adaptation to climate change (Sandring and Ågren, 2009; Keller et al., 2012). In this study, we found that in this perennial herb, the candidate genes associated with reproductive development and flowering were mainly significantly related to precipitation and aridity index (Table 3; Supplementary Figure S11).

A few candidate genes were ambiguous in how they are associated with climate. For example, an *LRR* (Leucine-rich repeat) receptor-like serine/threonine-protein kinase gene, associated with precipitation, appears to be involved in plant-pathogen interaction and developmental control (Afzal et al., 2008). Precipitation and moisture availability have a positive impact on the dispersal and infection success of phytopathogens (Swinfield et al., 2012; Milici et al., 2020), and pathogen recognition could be vital for plants in a mesic climate (Ahrens et al., 2019).

Overall, the results indicate that adaptation to climate is polygenic, potentially involving multiple adaptive mechanisms (Jordan et al., 2017; Dudaniec et al., 2018; Ahrens et al., 2019). Although the genes we identified only form a part of the broader adaptive evolutionary processes, we can take advantage of these patterns as a proxy to generate spatial patterns of adaptive variation and improve future management strategies. Future studies, based on high-quality genomic sequencing, knockout mutations and common garden experiments, are in need to validate the identified candidate gene regions in this study (Rellstab et al., 2015).

### 4.4 Population-level risk of future climate change

The influence of future shifts in climate across species’ distribution range is mediated by the collective potential of adapted populations to climate and/or migration in response. But few studies integrated intra-specific adaptation and migration when predicting how species responses to climate change (but see Sang et al., 2022). In this study, we followed a novel approach presented in Gougherty et al. (2021) to assess the contribution of *in situ* adaptation versus migration by simultaneously calculating three metrics of genomic offset. Overall, it is predicted that the genomic offsets are highest in central-north part of the species’ range, suggesting that there are no extant populations throughout the distribution range preadapted to the future climate in this area. Moreover, the effect of local climate shifts in this area cannot be mitigated by movement of populations to more suitable climate conditions, since the distances to locations that can minimize future maladaptation were predicted to exceed 3,000 km (largest within the range we assessed) that realistically is not reachable given the seed and pollen dispersal limitations. On the other hand, the genomic offset (vulnerability) of *T. hemsleyanum* was generally low in the southern (trailing) edge, which is in contrast with some theoretical work (Hampe and Petit, 2005). These patterns partially reflect the fact that the influence of temperature is secondary to that of precipitation/moisture condition in promoting the adaptive differentiation of loci we investigated, since precipitation in the coldest quarter is the most important climate variable in our GDM models (Supplementary Figure S7). The dominant effect of winter precipitation probably results from the involvement of a portion of candidate SNPs in drought response and/or phenological events in cool season. Future projection of precipitation predicted that the most notable change (a deceasing trend) of winter precipitation within our sampling area will occur in the middle reaches of the Yangtze river basin, especially along the mid-altitude mountainous region (Bucchignani et al., 2014; Bao et al., 2015) that largely corresponded to the central-north part of the species’ range. In addition to the winter precipitation decrease, the rising winter temperature will lead to increasing evapotranspiration, and exacerbate soil moisture losses. As predicted by high-resolution climate simulations (Yin et al., 2015; Ma et al., 2019), mutual reinforcement of these effects would induce a shift towards greater aridity in this humid region (aridity index increase ∼20% in 2070–2099) that populations may have not undergone in the recent past, thus rendering high adaptive offsets in central-north part of the range. Perhaps as a consequence of this, we observed significant associations of local and forward offsets not only with shifts in BIO19 and PETs (two most predictive variables in GDM), but with shifts in aridity index (Supplementary Figure S9). Besides, a general lack of correlation was observed between local/forward offset and the underlying pattern of population structure. This finding suggested that the genomic offsets in our focal species mainly reflect the influence of climate adaptation instead of the expected shifts in neutral variation.

Besides, our results further revealed substantial difference in the direction of migration that populations would follow to mitigate maladaptation to future climate challenges, and such pattern has been reported by several recent studies (Shaw, 2018; Gougherty et al., 2021). While most locations in the contemporary species range showed an overall northward shift, GDM indicated diverse population-level trajectories especially along the northwestern range edge (Figure 6B). Future climate projections by multiple general circulation models consistently discovered that subtropical China may experience an enhancement of spatial heterogeneity in precipitation and an expansion of arid regions (TCNARCC, 2011; Zhao and Wu, 2014). Since precipitation and PET related factors were the most significant predictors for GDMs, we propose that future shifts in precipitation regimes and moisture availability may account for some of the non-northward dispersal of *T. hemsleyanum* in our predictions.

Local populations vulnerable to future climate may occupy substantial unique adaptive genetic resources, thus special conservation and management efforts could be sensible as an insurance against such future genetic losses. For example, in central-north part of the range where *T. hemsleyanum* populations demonstrated maladaptation, we propose assisted gene flow strategy that involves the translocation of genotypes preadapted to future climate scenarios, particularly introducing from the moist and warm regions in southern margin of the mainland China. The genome-informed assisted gene flow can be beneficial for threatened species, as it may increase adaptive potential and alleviate inbreeding depression by introducing and increasing the frequency of adaptive alleles (Browne et al., 2019).

The reliability of genomic offsets as metrics of climate maladaptation was confirmed by recent works using data from population trend surveys and common garden experiments (Bay et al., 2018; Fitzpatrick et al., 2021). But it should be noted that the assumptions of future maladaptation made here is based on genomic SNP data, which does not account for alternatives (beyond allelic changes) for continued adaptation provided by epigenetic and expression changes, and phenotypic plasticity (Kenkel and Matz, 2016; Gao et al., 2022). Furthermore, we recognize that although we adopted GDMs to predict the genomic vulnerability to climate change, the genomic complexity of polygenic climate adaptation, for example, genetic redundancy and pleiotropy, have not been accounted here. It would be valuable to integrate quantitative genetics and systems biology methods to validate the current genotype–environment interactions and improve the prediction of species’ response to future climate challenges.

## Data Availability

The datasets presented in this study can be found in online repositories. The names of the repository/repositories and accession number(s) can be found below: https://www.ncbi.nlm.nih.gov/sra/, PRJNA922859.
