# Supplementary material for: Using landscape genomics to assess local adaptation and genomic vulnerability of a perennial herb Tetrastigma hemsleyanum (Vitaceae) in subtropical China
Source: Front Genet. 2023 Apr 18;14:1150704. doi: 10.3389/fgene.2023.1150704 (PMC10151583; doi:10.3389/fgene.2023.1150704)
Supplement: Supplementary file 1 [file DataSheet2.docx]

**Supplementary Figures**


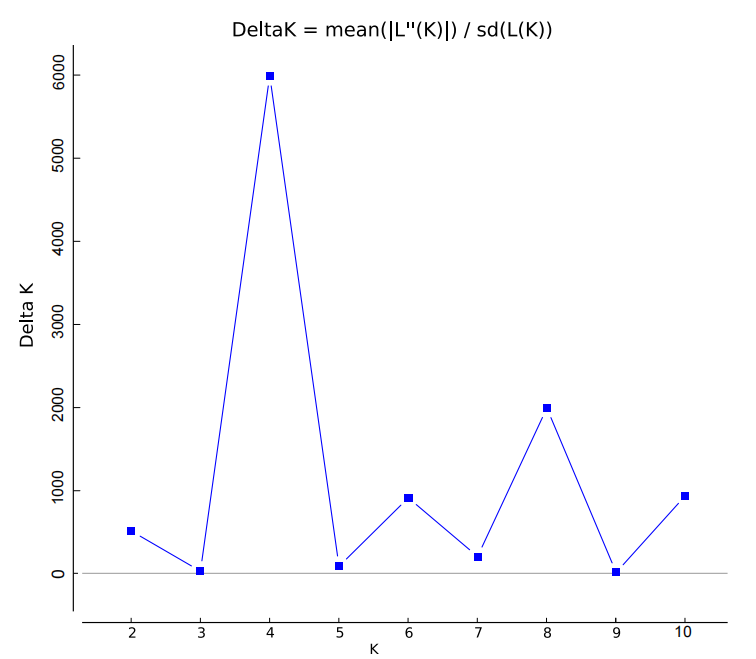


**Figure S1.** Bayesian clustering results of the STRUCTURE analysis for 24 populations of *Tetrastigma hemsleyanum* from subtropical China. Number of clusters (*K*) was varied from one to ten in ten independent runs. The corresponding ∆*K* statistics were calculated according to Evanno et al. (2005).


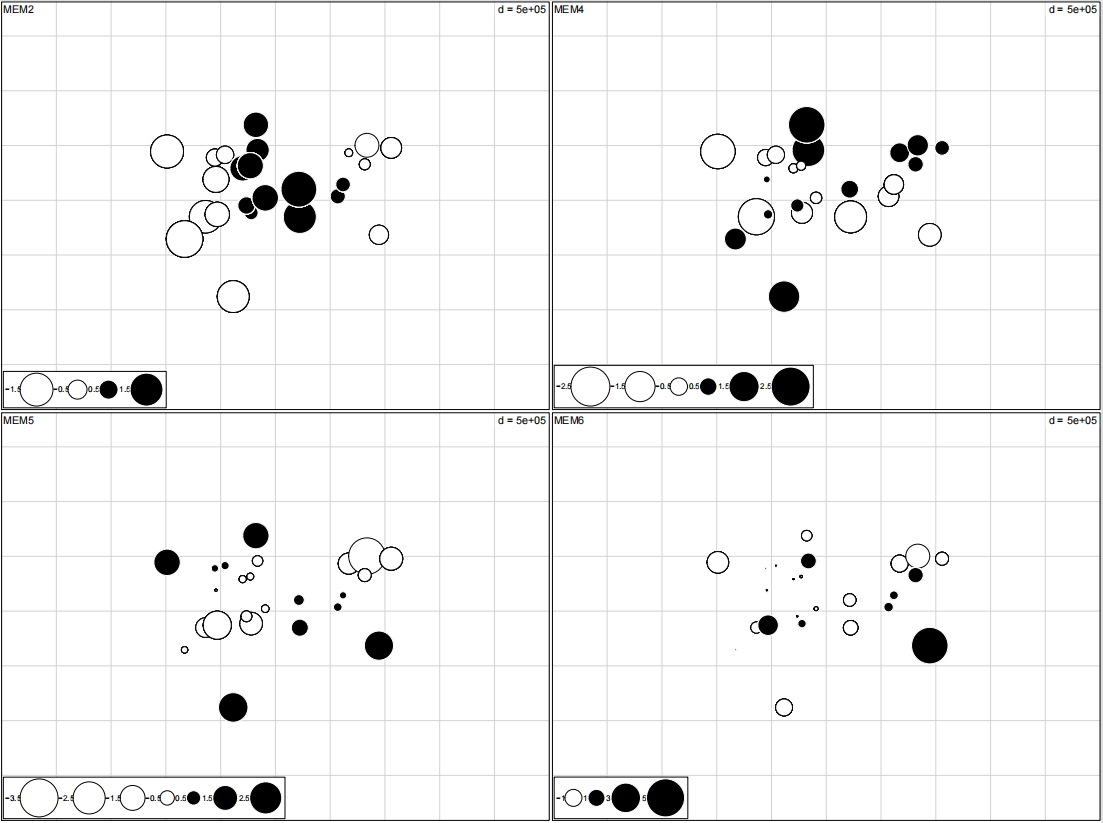


**Figure S2.** Bubble plots of the 4 dbMEM variables identified as significant. MEM2 and MEM4 variables describe broad patterns of spatial structure and MEM5, MEM6 describe fine-scale spatial variation. Color and size of the points correspond to the sign (+ or -) and magnitude of the dbMEM variables, respectively.


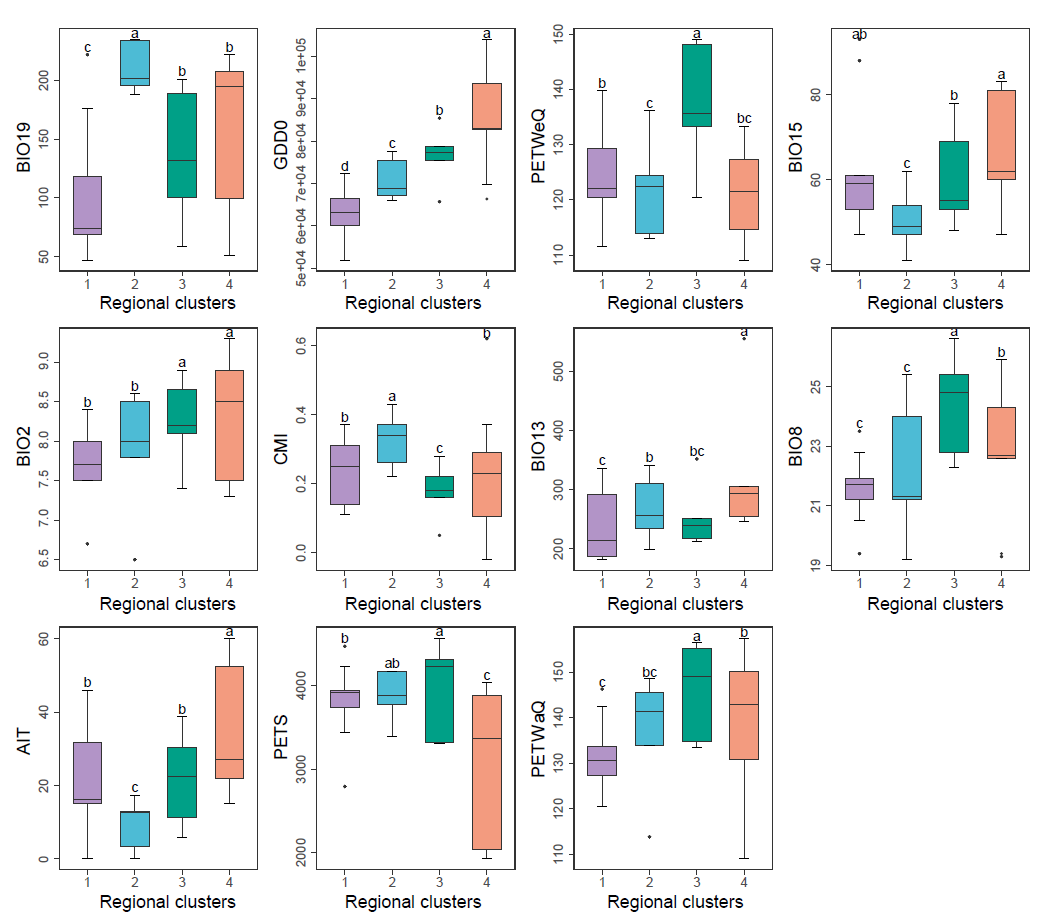


**Figure S3.** The variation of climate parameters by regional genetic cluster identified by STRUCTURE analysis. Colors correspond to genetic clusters identified in Figure 1. All panels were significant based on ANOVA (*P* < 1×10^-8^). Tukey HSD post-hoc comparison results are shown as letters above each box plot.


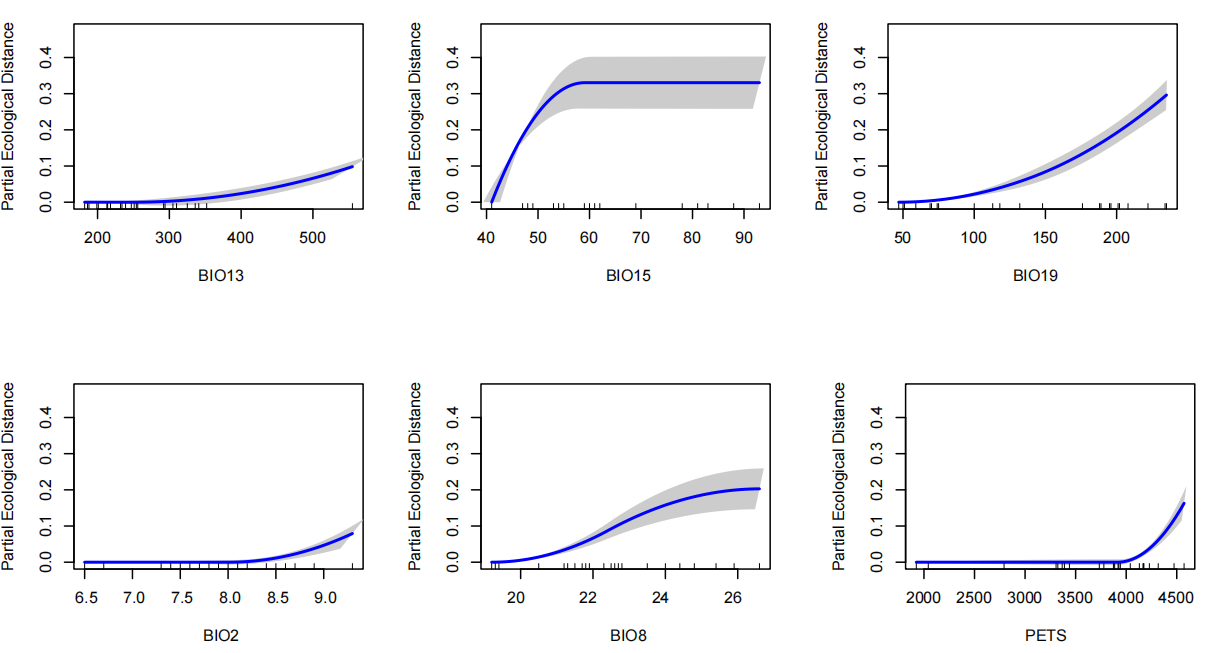


**Figure S4.** GDM spline fits for each climate predictor based on all SNP loci. The maximum height of each curve indicates the total amount of change in allele frequencies associated with that predictor (variable importance). The shape of each curve indicates how the rate of change in allele frequencies varies along the predictor gradient. To test the robustness of predictive I-splines, models were rerun with 10% of sampling locations removed in each data set. Each subgraph showed the range of spline fits among the 100 jackknife replicates (grey) and the full data (blue).


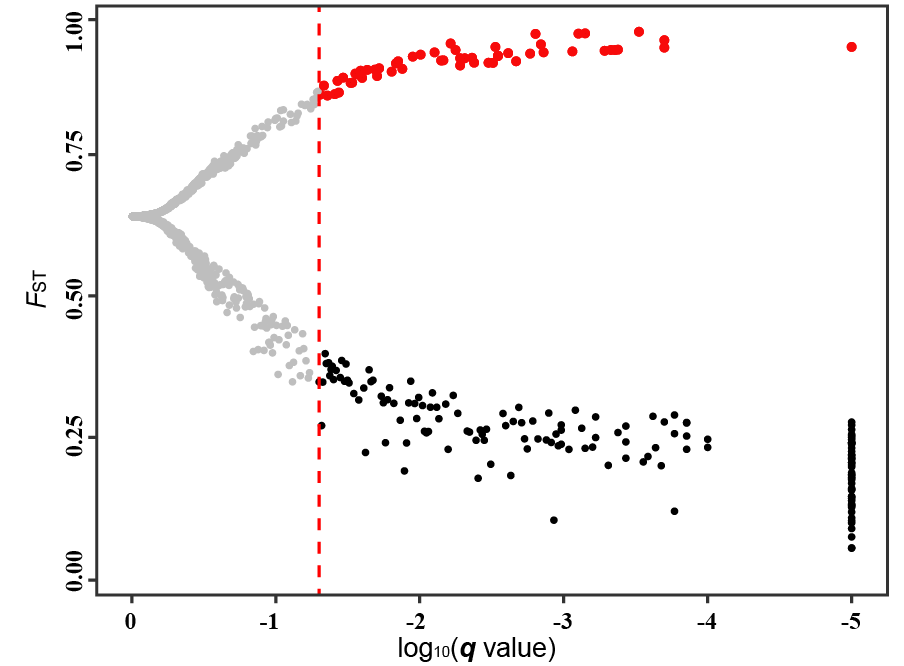


**Figure S5.** Identification of outlier loci putatively under selection in *Tetrastigma hemslyanum* populations using BayeScan for 30,252 SNPs. The vertical lines indicate the decision factor to determine selection in base-10 log scale log10(*q*-value) using a false discovery rate (FDR) of 0.05. Each dot represents a SNP. The dots to the right of the vertical lines represent the SNPs with outlier *F*_ST_ values. Red dot: outliers identified under diversifying selection; Black dot: outliers identified under balancing selection.


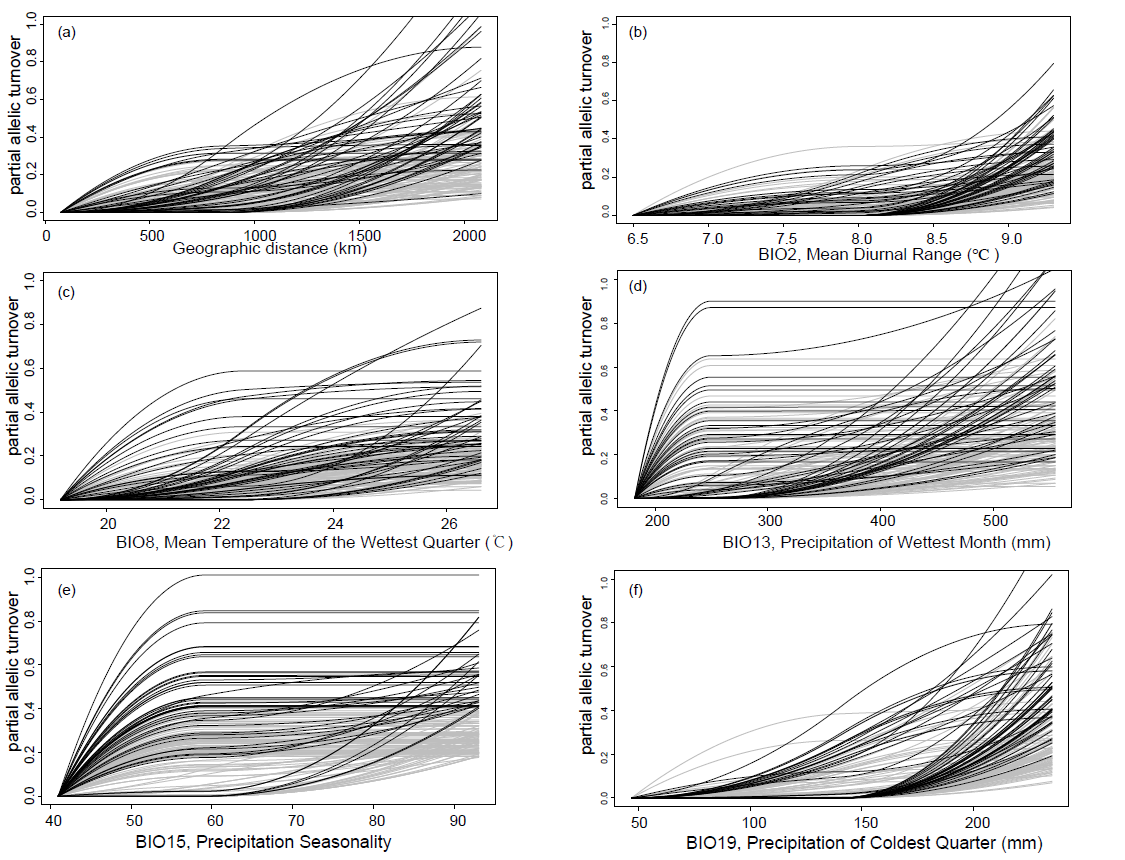


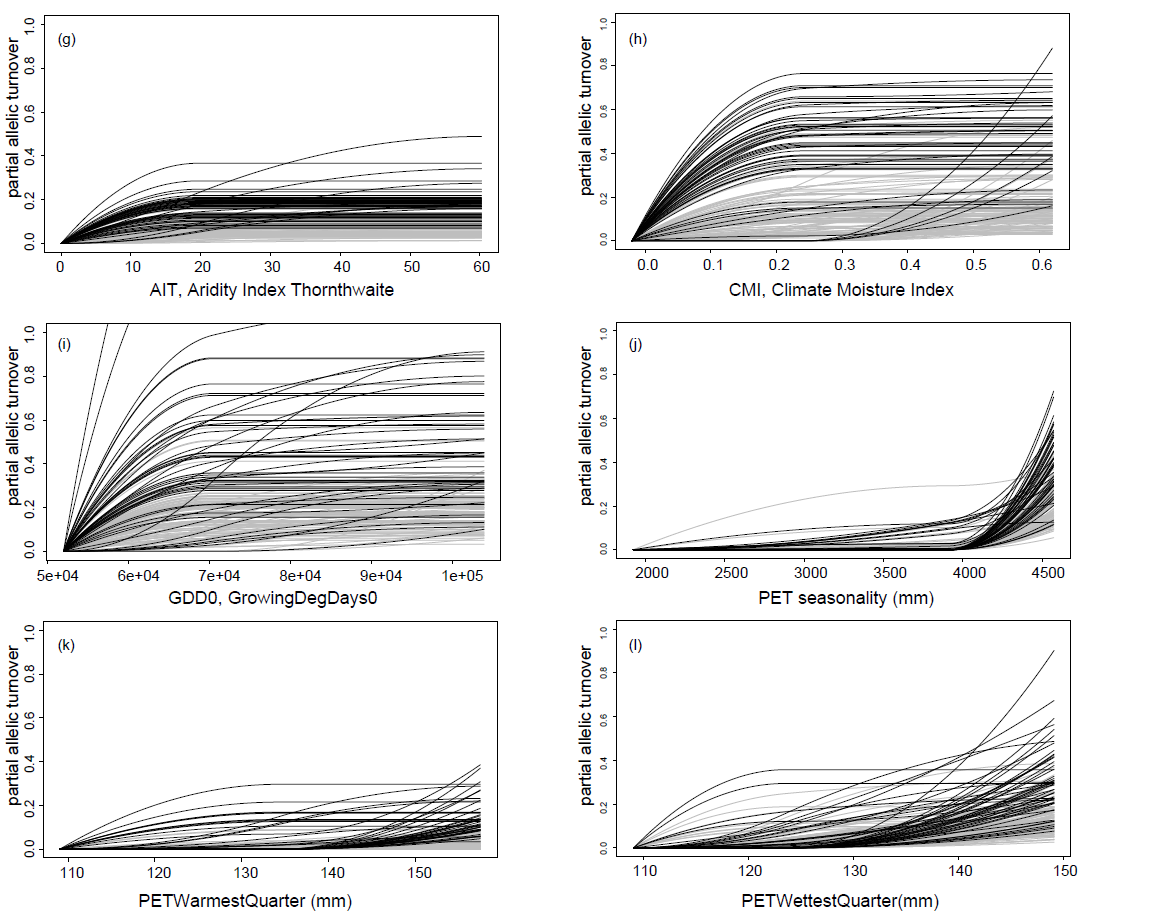


**Figure S6.** SNP-specific GDM results for the top 150 candidate SNPs (grey) and top 50 candidate SNPs (black) with the greatest partial allelic turnover in response to geographic distance and each climate variable (See Table S2 for variable abbreviations).


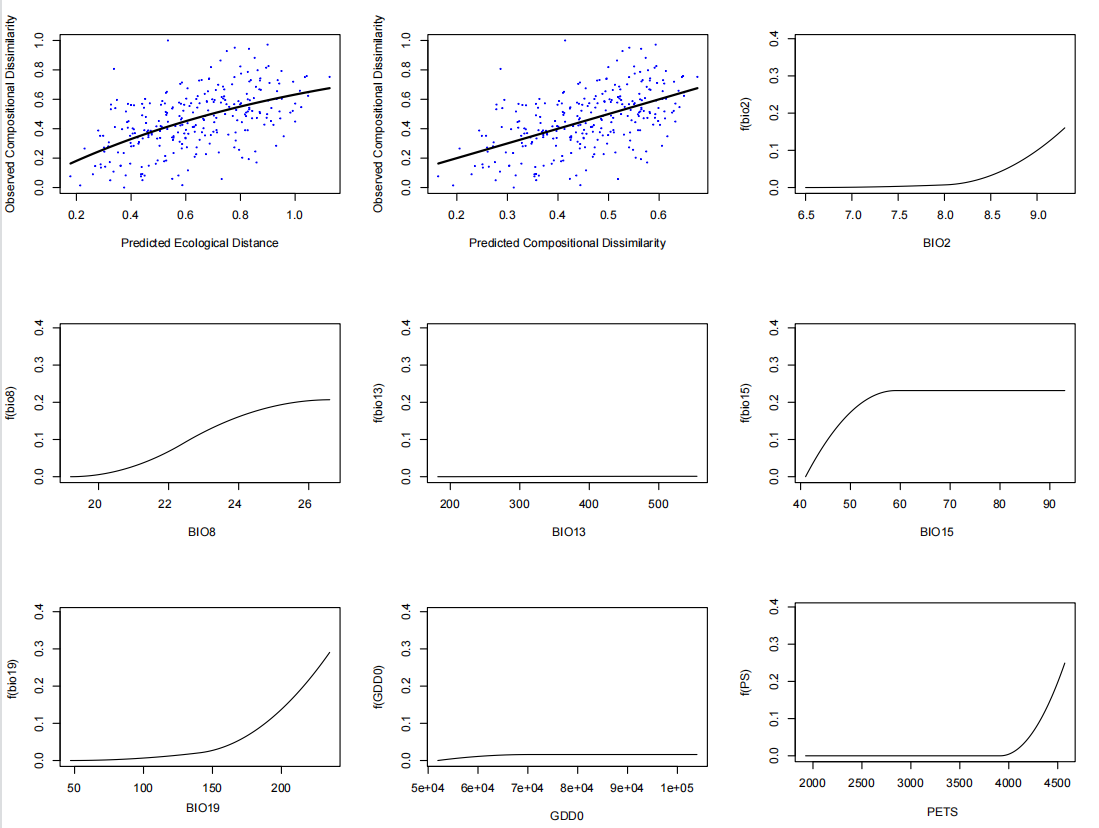


**Figure S7.** GDM fit and spline fits for geographic distance and each climate predictor (see Table S2 for variable abbreviations) based on putative adaptive loci identified by PD and EA methods. The maximum height of each curve indicates the total amount of change in allele frequencies associated with that predictor (variable importance).


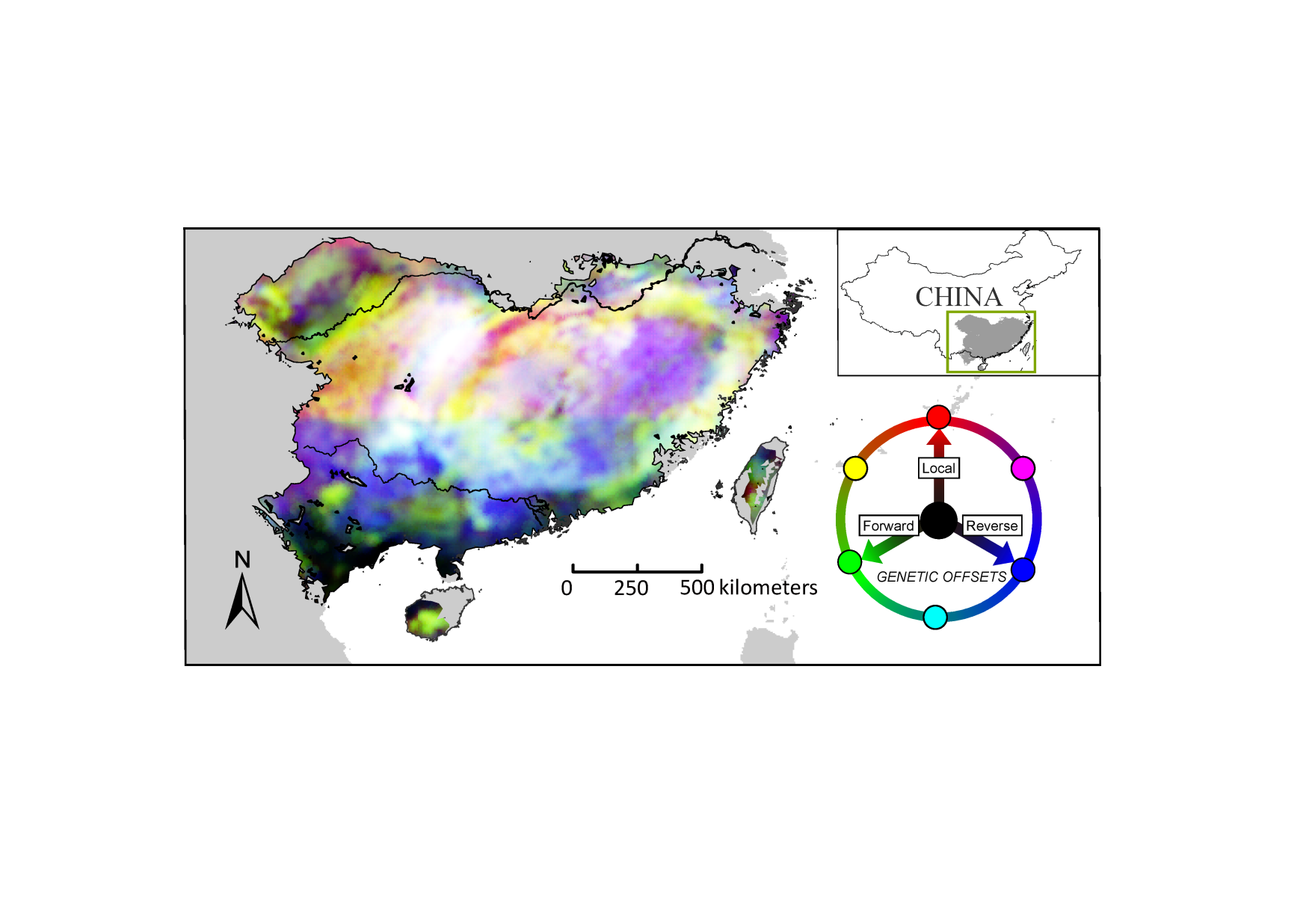


**Figure S8.** RGB map of local (red), forward (green) and reverse (blue) offsets estimated from GDMs across the distribution range of *T. hemsleyanum* for 2070 and SSP370 based on candidate adaptive genic SNPs. Cells with relatively higher values (higher expected vulnerability to climate change) along each of three axes were represented by brighter colors (closer to white). The individual band image creating this map are shown in Figure 6.


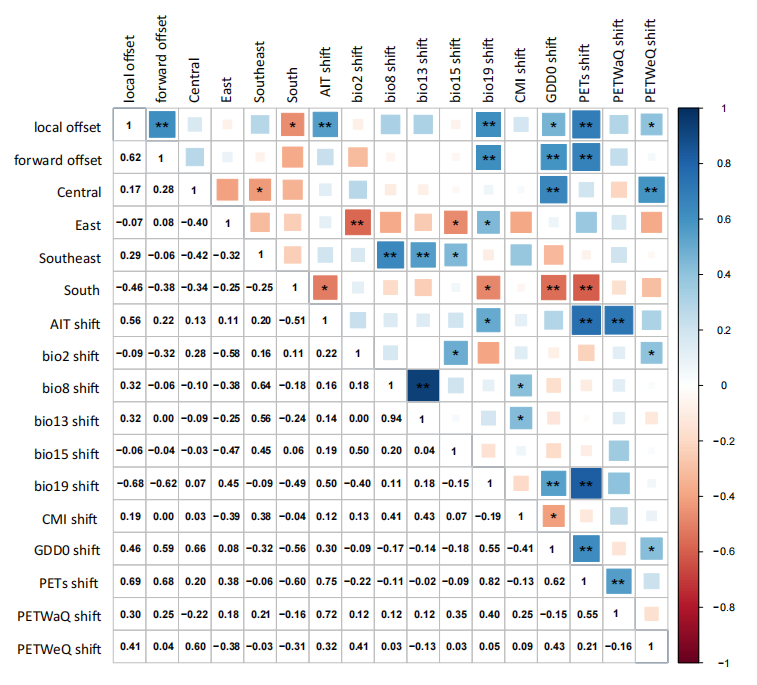


**Figure S9.** Spearman rank correlation between local and forward offsets from GDM, climatic shifts, and ancestry coefficients. Climatic shifts were calculated as future climate (2070, SSP370) minus current climate, for each climate variable. Ancestry coefficients represent the relative affiliation of each population to four genetic clusters (‘Southwest’, ‘Central’, ‘South’ and ‘East’ clusters).


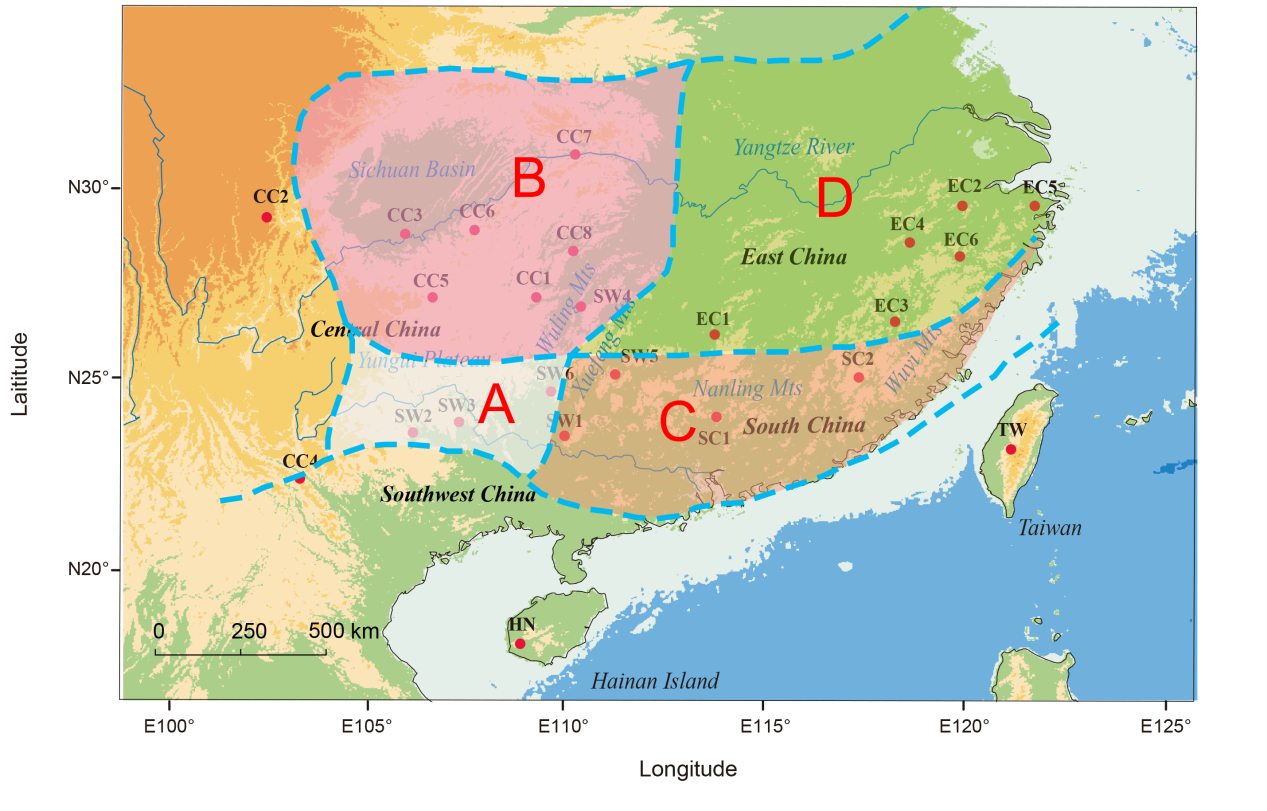


**Figure S10.** Four main floristic divisions (A–D) in Subtropical China according to Wu & Wu (1998). A. Yunnan-Guangxi-Guizhou (corresponding to Southwest China; mainly limestone vegetation of mixed forest with very diverse elements common to the Tongking Bay and the adjacent Yunnan Plateau); B. Central China (same as East China, but with more deciduous woody endemics and various conifers and taxads); C. South China (mainly evergreen pine-oak forest with many tropical elements); D. East China (mixed deciduous and evergreen broad-leaf forest). The red dots represent extant occurrence points.
